# A Mathematical Model for DC Vaccine Treatment of Type I Diabetes

**DOI:** 10.3389/fphys.2019.01107

**Published:** 2019-09-06

**Authors:** Blerta Shtylla, Marissa Gee, An Do, Shahrokh Shabahang, Leif Eldevik, Lisette de Pillis

**Affiliations:** ^1^Mathematics Department, Pomona College, Claremont, CA, United States; ^2^Mathematics Department, Harvey Mudd College, Claremont, CA, United States; ^3^Institute of Mathematical Sciences, Claremont Graduate University, Claremont, CA, United States; ^4^Aditx Therapeutics, Inc., Loma Linda, CA, United States

**Keywords:** type I diabetes, tolerogenic dendritic cells, regulatory T cells, macrophage clearance, immunotherapy, mathematical model, treatment simulation

## Abstract

Type I diabetes (T1D) is an autoimmune disease that can be managed, but for which there is currently no cure. Recent discoveries, particularly in mouse models, indicate that targeted modulation of the immune response has the potential to move an individual from a diabetic to a long-term, if not permanent, healthy state. In this paper we develop a single compartment mathematical model that captures the dynamics of dendritic cells (DC and tDC), T cells (effector and regulatory), and macrophages in the development of type I diabetes. The model supports the hypothesis that differences in macrophage clearance rates play a significant role in determining whether or not an individual is likely to become diabetic subsequent to a significant immune challenge. With this model we are able to explore the effects of strengthening the anti-inflammatory component of the immune system in a vulnerable individual. Simulations indicate that there are windows of opportunity in which treatment intervention is more likely to be beneficial in protecting an individual from entering a diabetic state. This model framework can be used as a foundation for modeling future T1D treatments as they are developed.

## 1. Introduction

Type I diabetes (T1D) is a chronic disease, often diagnosed in early childhood, that is characterized by an inability to regulate levels of glucose in the blood. Managing T1D requires daily injections of the hormone insulin. Even with proper management, individuals with T1D are at higher risk for serious health issues, including vision problems as well as heart, kidney, and nerve disease (Isley and Molitch, 2005). The causes of T1D are still not completely understood, but it is believed to be the result of a combination of environmental and genetic factors (Freiesleben De Blasio et al., 1999).

Type 1 diabetes is characterized by chronic elevated blood-glucose levels. These elevated glucose levels result from an abnormal auto-immune attack on insulin-producing β-cells in the pancreas. Cells in the body, such as fat, blood, and muscle cells, respond to insulin by absorbing glucose from the blood, thus lowering blood glucose levels. When insulin-producing pancreatic β−cells are damaged via an auto-immune attack, normal insulin production is disrupted, and blood glucose rises above healthy levels.

Specific clones of cytotoxic T-cells are thought to be the major culprit for this autoimmune response that invades the pancreatic islets of Langerhans (Varela-Calvino et al., 2017). However, many other types of immune cells are implicated in facilitation of the onset of disease. For example, Nerup et al. (1994) proposed that while the adaptive immune system does play a role in late stages of β-cell destruction, initial attacks are the result of macrophages secreting cytokines that are toxic to β-cells (Nerup et al., 1994). Dendritic cells (DC), which participate in antigen presentation, can contribute to initial onset and further development of the disease (Price and Tarbell, 2015). In non-obese diabetic (NOD) mice in particular, DCs appear in the pancreatic islets early on (3–4 weeks) and present self-peptides to auto-reactive T cells (Calderon et al., 2011; Carrero et al., 2016).

The non-obese diabetic mouse (NOD) has been an invaluable animal model system for the study of T1D, since in humans this disease is difficult to study non-invasively. In female NOD mice the timeline of the disease can be structured in stages. Specifically, as early as 2 weeks after birth immune cells infiltrate the pancreatic islets, at the 2–5 weeks mark macrophages and dendritic cells appear (Carrero et al., 2016), and at 6–10 weeks CD4+ and CD8+ T-cells engage with islets (Dahlén et al., 1998) (although CD8+ cells that are weakly insulin specific can emerge as early as 4–5 weeks; Trudeau et al., 2003). In NOD mice diabetes occurs when 95% of the β-cells are destroyed. Although incidence of T1D and time of onset can vary among different NOD mouse populations, typically 70–90% of female NOD mice develop T1D by 30 weeks of age, depending on strain, environment, and other factors (Alanentalo et al., 2010; Chaparro and DiLorenzo, 2010; King and Sarvetnick, 2011; The Jackson Laboratory, 2019). Prior to T1D onset when immune cells invade the islets, a general inflammatory state, referred to as insulitis, emerges. Interestingly, while almost all NOD mice develop insulitis, many do not develop T1D. A variety of genetic and environmental factors correlate with T1D. There are a few immune deficiencies that are thought to aid in the development of T1D in NOD mice, which in turn could mimic the factors that lead to T1D in humans. We highlight a few key NOD T1D related deficiencies that we examine closely in this paper. First, as part of normal development in neonatal rodents (both NOD mice and non-diabetes-prone Balb/c mice), the pancreas undergoes structural changes due to a high rate of apoptosis of β-cells during weaning, referred to hereafter as the *apoptotic wave* (Trudeau et al., 2000). Secondly, experiments have shown that macrophages from NOD mice have defective phagocytosis (O'Brien et al., 2002; H. et al., 2003; Maree et al., 2005). In addition, there is evidence that NOD mice have an IL-2 defect, which most likely affects proliferation and survival of Tregs (Tang et al., 2008), and in fact, T1D has been shown to be accelerated in Treg-deficient NOD mice (Feuerer et al., 2009). These factors collectively indicate that macrophages set the stage for the disease, and that Tregs are required to prevent disease progression. Antigen presenting cells, such as dendritic cells, can play a role in an important connection between macrophage engulfment and Treg expression (Creusot et al., 2014).

Understanding all the elements and complex interactions that contribute to differing immune system responses continues to be an active area of investigation. How cells die and how cell death and immune dynamics interact is a critical piece of the immune response puzzle. As discussed in Green et al. (2009), there are multiple varieties of cell death (including apoptosis, autophagic cell death, necrosis, secondary necrosis, pyropotosis, and mitotic catastrophe), and the conditions under which cells die (including cell death pathway, timing, and location) will trigger different cascades of events that can ultimately lead either to an immunogenic (inflammatory) or tolerogenic (anti-inflammatory) immune response. For example, it is generally understood that normal physiological cell death (apoptosis) contributes to tolerogenic immunity, whereas pathological cell death (necrosis) triggers an immunogenic (inflammatory) response. There is evidence that there are some exceptions to these categorizations, but for the sake of the model-building that we will do in this paper, we will adopt this simplified framework (i.e., apoptosis → tolerogenic response and necrosis → immunogenic response). For our purposes in this paper, we focus on the role that an engulfed dying cell has on DC activity. Specifically, the state of an engulfed cell (i.e., whether it is apoptotic or necrotic) in our framework dictates whether a DC will present an immunogenic (“fight”) signal that increases the action of effector T cells, or a tolerogenic (“stand down”) signal that increases the effects of regulatory T cells. We highlight that this function-based DC classification omits many details that are known about categorization of DC subsets, based on, for example, origin, phenotype, or function (Collin et al., 2013). For example, Steinman (1991, 2012), Banchereau and Steinman (1998), and Steinman et al. (2000, 2003) proposed a framework that broadly categorized monocyte-derived DCs as either immature or mature. In this framework, once DCs are resident in tissue, they are continually active in either immature or mature states. Immature DCs are more likely to be tolerogenic (that is, they contribute to the cascade of events that leads to an increase in the tolerogenic response). Once an immature DC is exposed to a danger signal (from a necrotic cell, for example), the DC matures, and is then more likely to contribute to an immunogenic response. Thus, we categorize DCs as either tolerogenic (and refer to these as tDCs), or immunogenic (simply referred to as DCs), with the understanding that tDCs are more likely to be immature, and DCs are more likely to be mature.

A number of mathematical models have been created to explore various hypotheses regarding key factors in T1D onset and progression (c.f. Nelson et al., 2009; Shoda et al., 2010; Jaberi-Douraki et al., 2014). One hypothesis referred to as the “Copenhagen model" in Freiesleben De Blasio et al. (1999) postulated that differences in macrophage engulfment rates can be sufficient to result in autoimmunity. While, Freiesleben De Blasio et al. (1999) performed preliminary mathematical analysis of this model, the results presented were primarily qualitative, and did not investigate the model for biologically grounded parameters of NOD mice. Motivated by the Copenhagen model, Marée et al. (2006) performed a quantitatively rigorous evaluation of the earlier, preliminary modeling; the key idea they tested was that NOD mice have a diminished ability to clear apoptotic β-cells, which then become necrotic. The core of their model relied on bistability between a healthy and a disease state, a transition which could be achieved with the help of variations of macrophage clearance rates. The role of T cells was then subsequently investigated first in Alexander and Walh (2011) where they studied a generic model of autoimmune disease that could be controlled by Tregs. Magombedze et al. (2010) extended the Maree et al. model to include generic Treg control of the autoimmune response. In Mahaffy and Edelstein-Keshet (2007), a mathematical model with activated, memory, and effector T cells was examined in the context of late disease progression when cyclic fluctuations in the levels of T cells in the blood were observed by Trudeau et al. (2003). Khadra et al. (2009) followed up with a simplified model that tracked various T cell clones with a primary focus on understanding the role of low avidity memory T cells that can shield β-cells, thereby generating a bistable system including a healthy and a diseased state. More recently, Jaberi-Douraki et al. (2015) examined the role of ER stress in β-cell death, whereas Moore and Adler (2016) used a model with many immune populations to explore the role of viral infection in the rate of progression of T1D in NOD mice. While all the above models have used nonlinear ODEs, recently Wedgwood et al. (2016) proposed an agent based spatial model using data from human pancreata collected close to the onset of T1D, with particular focus on the mechanisms underlying the development of insulitis in pancreatic islets.

In this paper, we propose a nonlinear ordinary differential equation mathematical model that explores the role of tolerogenic DCs in the initiation and progression of T1D in NOD mice. Our model relies on previous modeling strategies, such as Marée et al. (2006), where the implementation of the Copenhagen model that accounts for differences in macrophage clearance rates could recreate the healthy and NOD mouse natural histories in the context of blood glucose levels. In contrast to the approach by Marée et al. (2006), our model includes healthy β-cell counts as well as APC (antigen presenting) and T-cell populations. The primary goal of our model is to examine the role played in disease development by DC cell populations that have both immunogenic and tolerogenic properties. While, Moore and Adler (2016) also included multiple classes of immune cells, such as tolerogenic DCs in the context of viral infection, in our case we are interested in the specific generation of tolerogenic DCs from normal β-cell apoptosis in the pancreas. At the same time, we only include two categories of T cells, such as effectors and regulatory cells. Our approach extends prior work but also provides a new framework for testing curative DC based vaccine therapies. This is based on recent attempts to use DC injection in both mice (Machen et al., 2004) and a Phase I clinical trial (Giannoukakis et al., 2011) that showed DC treatments to be safe for human subjects. DC therapies have shown tremendous promise in cancer treatment (reviewed in Palucka and Banchereau, 2013), however, in their immature state DCs can show tolerogenic properties that can lead to stimulation of Tregs, which can have beneficial effects in autoimmune disease and transplant rejection (reviewed in Creusot et al., 2014). Indeed, Machen et al. (2004) showed that a single injection of DCs into 5–8 week old female NOD mice could significantly delay T1D onset and abolish insulitis in NOD mice that did not develop diabetes. Interestingly, while a single unmodified DC injection into 4–8 week old NOD mice can delay disease onset, a single injection into 10–12 week old NOD mice does not have the same effect (Creusot et al., 2014) unless supplemented with IL-4. The primary two questions we seek to answer through modeling here are:

- What role do tolerogenic DCs play in the development of the disease in the presence of a β-cell apoptotic wave in NOD (diabetes prone) and Balb/c (non-diabetes-prone) mice?
- What role do the timing and dosing of tDC injections have on the ability of NOD mice to escape T1D onset?

We show that tolerogenic DCs do not significantly affect disease onset. Instead, slowed macrophage clearance rates are the primary drivers for NOD T1D development. The addition of DC clearance narrows the range of model bistability, but overall produces results in agreement with prior work of Marée et al. (2006). Second and most important, we show that dose timing is critical for prevention of T1D onset. Using various DC dosing schedules we show that injections administered too early or too late can prove ineffective, indicating a sweet spot for changing the course of disease. Our simulated injection studies show that Tregs have a primary role in helping revert disease and that nonlinearities can produce interesting counter-intuitive results. Our simulated dosing results are in qualitative agreement with Machen et al. (2004). Our mathematical model can be used as a first step in the study of the role of timing and dosing of DC injections in NOD mice.

## 2. Materials and Methods

### 2.1. Model Formulation

**Figure 1:**
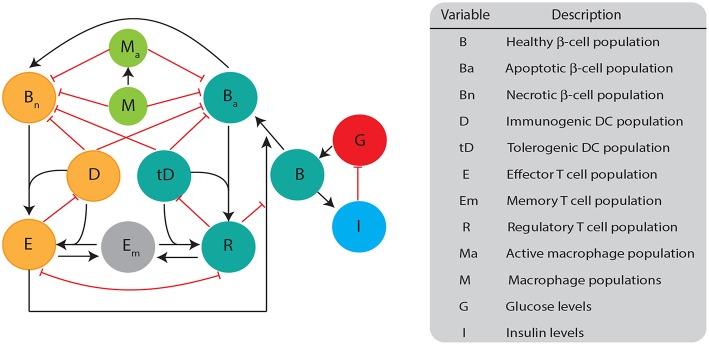
Diagram illustrating the biological quantities and interactions that are tracked in our single compartment model. β-cell populations are split into three categories: healthy population (*B*), apoptotic cells (*B*_*a*_), and necrotic cells (*B*_*n*_). Healthy β-cells are involved in glucose (*G*) and insulin (*I*) regulation, whereas the dying β-cells (apoptotic and necrotic) interact with immune cells. Dendritic cells are split into two categories: immunogenic DC (*D*) and tolerogenic DC (*tD*) based on their ability to engage an immunogenic or tolerogenic T cell response. DCs in our model are assigned into a particular category once they engulf either apoptotic or necrotic β-cells. Macrophages can either be in an activated (*M*_*a*_) or non activated form (*M*) based on their capacity to engulf β-cells. T cells are split into: regulatory T (*R*), effector T (*E*), or memory T (*Em*). All populations are measured in cells ml^−1^ except for *B* (mg), *G* (mg dl^−1^), and *I* (μ U).

The mathematical model we developed here consists of a single well mixed pancreatic compartment that includes various types of β-cells and immune cells, diagrammed in Figure 1. For the β-cell populations, we included healthy (*B*), apoptotic (*B*_*a*_), and necrotic (*B*_*n*_) populations, similar to Marée et al. (2006). For the immune cell populations, we restricted our attention to APC populations of macrophages and DCs, as well as T cell populations including effector, regulatory, and memory T cells. While it is likely that more immune populations are involved, our goal is to keep a simple framework in which two classes of DCs compete for activation of either effector or regulatory cells. All the model populations are listed in Figure 1.

There are specific target behaviors that we seek to capture in our model structure in order to effectively characterize the development of T1D, and to improve the accuracy of DC treatment simulations. As observed in Marée et al. (2006), there are differences in macrophage clearance rates between NOD and Balb/c mice that can lead to T1D in some mathematical models. Thus, our model is constructed to have the property that differences in macrophage clearance rates between NOD and Balb/c mice lead to differences in the development of the disease. For the macrophage population equations, we use the same formulation as the model examined in Marée et al. (2006), which captures interactions between resting/inactive (*M*) and active (*M*_*a*_) macrophages, apoptotic, and necrotic β-cells. The equations governing macrophage behavior are

(1) $$\begin{array}{l} \frac{d}{dt}M=J+(k+b)M_{a}-cM-f_{M}MB_{a}-f_{M}MB_{n}\\ \;-e_{1}M(M+M_{a}), \end{array}$$

(2) $$\begin{array}{l} \frac{d}{dt}M_{a}=f_{M}MB_{a}+f_{M}MB_{n}-kM_{a}-e_{2}M_{a}\left(M+M_{a}\right). \end{array}$$

Following Marée et al. (2006), in Equation (1) resting macrophages are recruited in the pancreatic compartment with constant influx rate *J*, whereas activated macrophages recruit additional resting macrophages into the compartment with rate *b*. In Equations (1)–(2), a resting macrophage becomes activated when it engulfs either a necrotic or an apoptotic β-cell with rate *f*_*M*_. In turn, activated macrophages convert back (or deactivate) into the resting population with rate *k*. Finally, both populations are decreased in response to macrophage crowding which could result in reduced macrophage entry into the pancreatic compartment with rate *e*_1_, *e*_2_, respectively.

Next, we incorporate the dynamics of healthy β-cells, glucose, and insulin in addition to their interactions with T cells with the following equation

(3) $$\begin{array}{l} \frac{d}{dt}B=\alpha_{B}K_{1}\left(G\right)B-\delta_{B}B-\eta_{e}\left(t\right)K_{2}\left(E,R\right)B-W\left(B,t\right). \end{array}$$

We assume that healthy β-cells are produced at a rate proportional to glucose levels *K*_1_(*G*). We employ the glucose dependent growth term derived in Equation (5.2) of Graham (2012)

(4) $$\begin{array}{l} K_{1}\left(G,G_{hb}\right)=\frac{G^{2}}{G^{2}+G_{hb}^{2}}. \end{array}$$

This term is similar to the glucose-dependent β-cell growth term of De Gaetano et al. (2008) (Appendix 1, Equation 6). This form postulates a slow saturating growth term with half max glucose level of *G*_*hb*_. In addition, healthy β-cells die at a constant rate δ_*B*_ due to a natural lifespan of the cells. There is evidence that effector T cells kill their targets by programming them to undergo apoptosis (Janeway et al., 2001). Thus, in the model, when β-cells encounter effector T cells, we will have them enter an apoptotic state with rate η_*e*_(*t*)*K*_2_(*E, R*). The T cell apoptosis term is given by the nonlinear saturating function

(5) $$K_{2}(E,R)=\frac{{\left(s_{E}E\right)}^{2}}{1+{\left(s_{E}E\right)}^{2}+{\left(s_{R}R\right)}^{2}},$$

which includes both a saturating effect in effector T cells and a slow down in β-cell apoptosis in the presence of regulatory T cells, similar to Moore (2015). The rate η_*e*_(*t*) captures the effectiveness of β-cell killing by effector T cells and it is an increasing function in time given by

(6) $$\begin{array}{l} \eta_{e}\left(t\right)=\eta +2\eta \left(1+\tanh\left(\alpha_{e}\left(t-\beta_{e}\right)\right)\right). \end{array}$$

A plot illustrating the functional form of this time-dependent rate is shown in Supplementary Figure S3. The rapidity of the ascent to the steady state maximum value in η_*e*_(*t*) is controlled by the parameters η, α_*e*_, and β_*e*_. This form is chosen so as to capture an increasing avidity of effector T cells against β-cells as a function of time. This may be because presumably more antigen becomes available with disease progression, thereby increasing the number of autoantigen-specific T cells (Trudeau et al., 2003; Krishnamurthy et al., 2006). The parameters α_*e*_, β_*e*_, which describe the rate at which effector T cell avidity increases over time, are estimated here from glucose data of Li et al. (2010). The term *W*(*B, t*) captures death of healthy β-cells due to a normal developmental apoptotic wave, as first postulated in Trudeau et al. (2000). We use a slightly modified term from Marée et al. (2006) with

(7) $$W(B,t)=.1wBe^{-{\left(\frac{t-9}{9}\right)}^{2}}$$

which encodes that the wave peaks at approximately 9 days corresponding to 10% of the β-cell population mass. The scalar multiplier *w* is a parameter we vary in order to control the magnitude of the wave in our simulations. We note that we do not incorporate new cell growth which occurs in the growing mouse pancreas. This assumption results in a more pronounced model β-cell mass decline in the presence of the apoptotic wave than what has been observed in experiments (Finegood et al., 1995). Unless otherwise stated, we set *w* = 0.75 in our model.

The model equations for apoptotic and necrotic β-cells populations are

(8) $$\begin{array}{l} \frac{d}{dt}B={\tilde{\delta }}_{B}B+{\tilde{\eta }}_{e}(t)K_{2}(E,R)B+\tilde{W}(B,t)-dB_{a}-f_{M}MB_{a}\\ \;-f_{M_{a}}M_{a}B_{a}-f_{tD}(D_{ss}-D)B_{a}-f_{D}DB_{a} \end{array}$$

(9) $$\frac{d}{dt}B_{n}=dB_{a}-f_{M}MB_{n}-f_{M_{a}}M_{a}B_{n}-f_{tD}(D_{ss}-D)B_{n}-f_{D}DB_{n}.$$

Apoptotic β-cells in Equation (8) are generated from healthy β-cells in three ways: regular cell turnover with rate ${\tilde{\delta }}_{B}$, in response to effector T cells killing with rate ${\tilde{\eta }}_{e}\left(t\right)$, and from the apoptotic wave. The ${\tilde{\delta }}_{B},{\tilde{\eta }}_{e}\left(t\right),\tilde{W}$ notation is used to mark the multiplication of each corresponding parameter with a $\frac{B_{conv}}{Q_{panc}}$ factor that allows unit conversion for *B* from milligrams into cells/ml to match the units of *B*_*a*_, *B*_*n*_; *Q*_*panc*_ stands for pancreas volume, whereas *B*_*conv*_ gives an estimate of number of β-cells per milligram using cell counts from Chintinne et al. (2012). Apoptotic β-cells decay into necrotic β-cells at the rate *d*, following estimates in Marée et al. (2006). A key new addition in our model compared to Marée et al. (2006) is that both apoptotic and necrotic β-cell populations are engulfed and cleared by tDCs and DCs as well as by resting and activated macrophages, with rates *f*_*tD*_, *f*_*D*_, *f*_*M*_, *f*_*Ma*_, respectively. While the rates of macrophage engulfment and β-cell clearance have been estimated (Marée et al., 2006), in this paper we will estimate DC clearance rates.

Glucose and insulin dynamics are modeled using the equations developed in Topp et al. (2000) with

(10) $$\begin{array}{l} \frac{d}{dt}G=R_{0}-\left(G_{0}+S_{I}I\right)G, \end{array}$$

(11) $$\begin{array}{l} \frac{d}{dt}I=\sigma_{I}K_{1}\left(G,G_{I}\right)B-\delta_{I}I. \end{array}$$

The equations are built on the assumptions that glucose enters the blood stream at a constant rate *R*_0_, leaves the bloodstream at a base rate *G*_0_*G*, and is taken up at an additional rate proportional to the presence of insulin, given by *S*_*I*_*IG*. Insulin is in turn produced at a rate proportional to the number of healthy β-cells, as well as the presence of glucose up to a saturation point parameterized by *G*_*I*_. Insulin decays at a natural rate δ_*I*_*I*.

Next, we incorporate DC cell dynamics with the following equations

(12) $$\begin{array}{l} \frac{d}{dt}D=f_{tD}B_{n}\left(D_{ss}-D-tD\right)+f_{tD}B_{n}tD-b_{DE}ED-\mu_{D}D \end{array}$$

(13) $$\begin{array}{l} \frac{d}{dt}tD=f_{tD}B_{a}\left(D_{ss}-D-tD\right)-f_{tD}B_{n}tD-b_{IR}RtD-\mu_{D}tD. \end{array}$$

For the DC and tDC equations we are assuming that there are three pools of DCs in the model compartment and we distinguish each category by its engulfed apoptotic cell status and specificity as follows: (1) *D*_*ss*_ corresponding to all types of DCs in the pancreas (2) *tD* corresponding to migratory and immature (tolerogenic) DCs that have engulfed an apoptotic beta cell and elicit a particular regulatory T cell response, (3) *D* corresponding to migratory and mature (immunogenic) DCs that have engulfed a necrotic beta cell and elicit a particular effector T response, (4) *iD* = *Dss* − *tD* − *D* corresponding to immature resident DCs (non-migratory) that have not engulfed some sort of dying β-cell. Immature resident DCs engulf necrotic or apoptotic β-cells at rate *f*_*tD*_. Note that the engulfment rate for these immature DCs is assumed to be the same as the engulfment rate of tolerogenic DCs, *f*_*tD*_, since tDCs are thought to be in an immature state compared to immunogenic DCs (Mackern-Oberti et al., 2015). In addition, tolerogenic DCs can convert into an immunogenic state upon engulfing a necrotic cell with rate *f*_*tD*_. DCs and tDCs are neutralized upon encountering effector and regulatory T cells with rates *b*_*DE*_ and *b*_*IR*_, respectively; this term includes both direct elimination of DCs by T cells (Yang et al., 2006) and indirect pathways (reviewed in Ronchese and Hermans, 2001). We highlight that this term is written using a simplifying assumption that DC and T cells engage exclusively in the pancreas due to the single compartment nature of our model. However, physiologically DCs are generated and lead to proliferation of T cells in different locations such as the spleen and or lymph nodes. In the interest of simplicity, we approximate these interactions into a single modeling term in the pancreas with a main goal of avoiding a scenario in which DCs and tDCs overcrowd the pancreas, leading to unrealistically high numbers of T cells. Finally, we assume that both types of DCs have a constant death rate, μ_*D*_.

We next discuss the T cell populations and interactions in our model. We incorporate three T cell populations, namely: effector (*E*), regulatory (*R*), and memory T cells (*Em*). The interactions between these three populations are described by

(14) $$\begin{array}{l} \frac{dE}{dt}=a_{E}\left(T_{naive}-E\right)+b_{P}\frac{DE}{\theta_{D}\;+\;D}-r_{am}E+b_{E}DEm-\mu_{E}ER, \end{array}$$

(15) $$\begin{array}{l} \frac{dR}{dt}=a_{R}\left(T_{naive}-R\right)+b_{P}\frac{tDR}{\theta_{D}+tD}-r_{am}R+b_{R}tDEm\\ \;-\mu_{R}ER, \end{array}$$

(16) $$\begin{array}{l} \frac{dEm}{dt}=r_{am}\left(E+R\right)-\left(a_{Em}+b_{E}D+b_{R}tD\right)Em. \end{array}$$

Our model equations follow closely the models of Ludewig et al. (2004) and DePillis et al. (2013) in order to quantify how T cells are produced. Specifically, the model developed in Ludewig et al. (2004) and DePillis et al. (2013) is for T cell production in the spleen, so we adapt to fit the context of the current model, where clearance and immune cell generation happen simultaneously. The first term in Equations (14, 15) represents a homeostatic naive T cell term that generates basal levels of effector and regulatory cells. The second term in Equations (14, 15) gives the DC-induced proliferation of effector or regulatory T cells, which we assume happens with equal rates *b*_*P*_ for both effector and regulatory T cells. We note that here we assume activation is only achieved through a specific DC or tDC interaction that requires constant contact between a DC or tDC and an effector or regulatory T cell, respectively. In Ludewig et al. (2004) and DePillis et al. (2013) these proliferating saturation terms are modeled with time delays to capture so called “pre-programming” of T cells for division and differentiation. In our case, since simulations extend for months, we assume that the delay is short in comparison and omit it. The specifics of T cell proliferation mechanisms in various modeling setups have been reviewed in Wodarz (2007)—we retain a simplified DC saturating term in our model indicating that proliferation is limited by the availability of DCs. Effector and regulatory cell interactions are incorporated with simplified mutual down-regulation terms with rates μ_*E*_, μ_*R*_ to incorporate the mutual inhibition of effector and regulatory T cells. We note that such a term aims to most directly incorporate the effects on immune response due to differences in effector vs. regulatory T cell numbers, however, physiologically each T cell population can be suppressed through a variety of direct and indirect pathways (Sakaguchi et al., 2008). Finally, our memory T cell population is shared by the effector and regulatory T cells; we have tested versions of the model where two separate memory cell classes are assigned for the effector and regulatory T cells and noted no significant model response changes. Thus, in the interest of simplicity we opted for a single memory T cell population.

### 2.2. Model Parameterization

We performed parameter fitting on published DC data in order to incorporate the DC effect on the development of T1D. The types of cells that DCs engulf play a role in what type of specific immune response they generate (reviewed in Green et al., 2009). To investigate how encounters between DCs, apoptotic β-cells, and necrotic β-cells contribute to an autoimmune response to pancreatic β-cells, we first determine the rates at which DCs engulf these cells. We describe these values as the “clearance rate” of each type of DC. Such values are currently missing from the modeling literature surrounding T1D. Thus, this section draws on experimental data collected in Albert et al. (1998) and stochastic parameter fitting techniques to model these interactions and find appropriate values for the clearance rates.

### 2.3. Experimental Setup and Data

The experiment of interest is described in Albert et al. (1998) in which human-derived macrophages, immature DCs, and mature DCs were observed as they phagocytosed apoptotic cells, and rates of engulfment and clearance were measured. In this experiment, macrophages and immature DCs were isolated from human subjects, and a portion of the immature DCs were stimulated to mature. Each cell type was then placed with isolated apoptotic cells that had not yet undergone necrosis, and were allowed to interact over the course of 4 h. Cells were added in a 1:1 apoptotic to phagocytic cell ratio. The key conclusion reached in Albert et al. (1998) was that macrophages and the two types of DCs appeared to phagocytose at different rates. This experiment provides us with information on the relationship between rates of phagocytosis for macrophages vs. phagocytosis rates for DCs and tDCs. Because the experiment in Albert et al. (1998) involved human rather than mouse immune cells, we do not directly use those clearance rate values for DCs, tDCs, or macrophages in our mouse model.Nonetheless, we need to make a reasonable approximation to the needed clearance rates, *f*_*D*_ and *f*_*tD*_ in mice. In order to find mouse rate approximations, we chose to make the assumption that although rates in mice and humans may differ, the ratios of the relative macrophage to DC and macrophage to tDC engulfment rates will be the same in mice as in humans. Thus, using measured mouse macrophage engulfment and clearance rates from Marée et al. (2006), and ratios of macrophage to DC and tDC engulfment and clearance rates in humans (as measured in Albert et al., 1998), we computed DC and tDC engulfment rates in mice to determine our clearance rate parameters *f*_*D*_ and *f*_*tD*_. For the purpose of carrying out the rate computations, we developed a reduced mathematical model that focused only on how each macrophage and DC population engulfed dying cells in the assay of Albert et al. (1998). Using this reduced model, we could employ mathematical parameter fitting techniques to extract the needed engulfment rates.

### 2.4. Simplified Interaction Model

We developed a simplified set of mass action equations to model the experimental setup. In Albert et al. (1998), there is no distinction made between active and inactive phagocytic cells so we assume that the rates reported for macrophages correspond to the rate of active macrophages in our model, and similarly the rates for immature and mature DCs correspond to the rates for tolerogenic and immunogenic DCs in our model. Based on these assumptions, we model each type of phagocytic cell in Albert et al. (1998) as engulfing with a single clearance rate that does not depend on how many cells it has already phagocytosed. Using these assumptions we develop the following governing equations, which can be used for all three types of phagocytic cells,

(17) $$\begin{array}{l} \frac{d}{dt}C=-g_{P}PC, \end{array}$$

(18) $$\begin{array}{l} \frac{d}{dt}P_{e}=g_{P}\left(P-P_{e}\right)C, \end{array}$$

where *C* represents the apoptotic cell population, *P*_*e*_(*t*) represents the population of phagocytic cells that have engulfed something since the beginning of the experiment, and *P* the total population of phagocytic cells. Note that *P*_*e*_, *P* are generic population terms that represent *M, D* or *tD* for each data set (see Table 1). The model is initialized with *C*(0) = *P* and *P*_*e*_(0) = 0. We note that we track *P*_*e*_ because we are interested in computing the percent phagocytosis as computed in the experimental data.

**Table 1:** State variables describing the cell populations relevant to the ODE model fit to data from Albert et al. (1998), as well as their meanings.

**Variable name**	**Units**	**Meaning**
*C*	cells ml^−1^	Apoptotic cell population
*P*_*e*_	cells ml^−1^	Phagocytic cell population
*M*	cells ml^−1^	Macrophage population
*D*	cells ml^−1^	Mature DC population
*tD*	cells ml^−1^	Immature DC population

We can solve the system to obtain the following solutions

(19) $$\begin{array}{l} C\left(t\right)=Pe^{-g_{P}Pt} \end{array}$$

(20) $$P_{e}(t)=P\left(1-e^{e^{\left(-g_{P}Pt\right)}-1}\right).$$

Percent phagocytosis, ρ is then given by ρ = *P*_*e*_(*t*)/*P* × 100%. We note that as *t* → ∞, $P_{e}\left(t\right)\to P\left(1-e^{-1}\right)$. This tells us that our steady-steady percent phagocytosis does not depend on the rate at which phagocytic cells engulf apoptotic cells. This presents a challenge in fitting this model to measurements in Albert et al. (1998) at larger values of time, because the experimental data indicates different steady state values for different cell types. However, we can fit this model to the behavior of each cell type at small time values. Furthermore, we are more interested in the transient behavior of the model, because this represents interactions that occur without the effects of a limited source of apoptotic cells. Because our full pancreas model assumes apoptotic cells are being produced in the system, we would expect behavior there to also not be limited by the apoptotic cell population. We chose to use data up to 1 h in our fitting, as this is the time frame in which there did not appear to be significant saturation of percent phagocytosis in the data.

### 2.5. Pancreatic Compartment DC Parameter Values

We fitted the parameters for the simplified model using the Maximum Likelihood Estimate (MLE), implemented using the Metropolis Monte Carlo Markov-Chain (MCMC) algorithm for this process, detailed in the Supplementary Information. Next, we used the parameter fits from the simplified model in order to determine rates for DCs and tDCs in the full pancreas model as follows. We assumed that ratios between rates for macrophages, immature DCs, and mature DCs were the same in humans and mice, so we could use the ratios of the parameter values found above to determine parameters for a mouse model. Then using the mouse macrophage clearance rate estimated in Marée et al. (2006), corresponding to *f*_*M*_*a*__, we computed the rates at which immature and mature DCs phagocytose by scaling *f*_*M*_*a*__ by the appropriate fitted ratios. Let *f*_*D*_ and *f*_*tD*_ represent the clearance rates of immunogenic and tolerogenic DCs, respectively, then

(21) $$\begin{array}{l} f_{D}=f_{M_{a}}\frac{g_{D}}{g_{M_{a}}}=5.49\times 10^{-2}f_{M_{a}} \end{array}$$

(22) $$\begin{array}{l} f_{tD}=f_{M_{a}}\frac{g_{tD}}{g_{M_{a}}}=3.82\times 10^{-1}f_{M_{a}}. \end{array}$$

In order to study the effects of reduced macrophage clearance in isolation, we assumed that DCs and tDCs in NOD mice were not impaired, meaning that DC and tDC clearance rates in our model were assumed to be the same for NOD and Balb/c mice. This corresponds to scaling *f*_*D*_, *f*_*tD*_ from the rate that active macrophages clear in healthy Balb/c mice, as shown in Equations (21)–(22). In addition, we assumed that necrotic cells were phagocytosed at the same rate as apoptotic β-cells.

All parameter values used in our model and corresponding sources are outlined in the Appendix 1.

## 3. Results

### Simulating T1D Initiation

**Figure 2:**
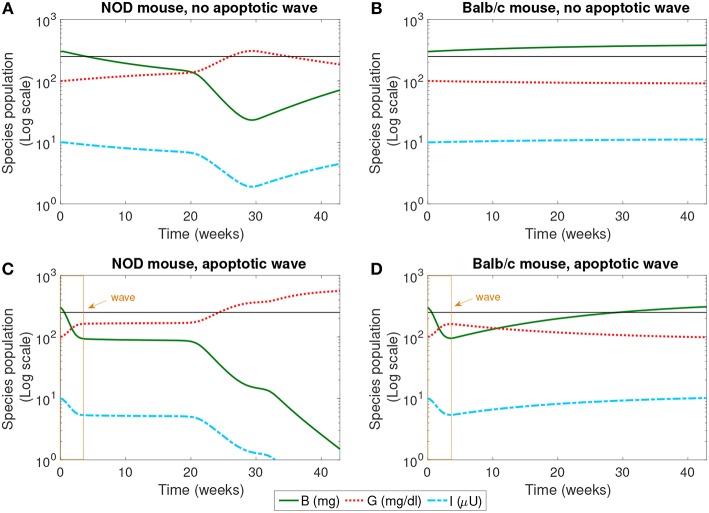
The populations of healthy β-cells (*B*), glucose (*G*), and insulin (*I*) simulated for 40 weeks, according to the mathematical model. We compare the system in the presence and absence of an early wave of apoptosis, and between NOD and Balb/c mice. Multiple glucose readings above 250 mg/dl (shown by the horizontal line) indicate the progression of T1D. The only system that crosses this threshold is the NOD mouse in the presence of the apoptotic wave **(C)**. **(A)** NOD mouse, no apoptotic wave, **(B)** Balb/c mouse, no apoptotic wave, **(C)** NOD mouse, apoptotic wave, and **(D)** Balb/c mouse, apoptotic wave.

**Figure 3:**
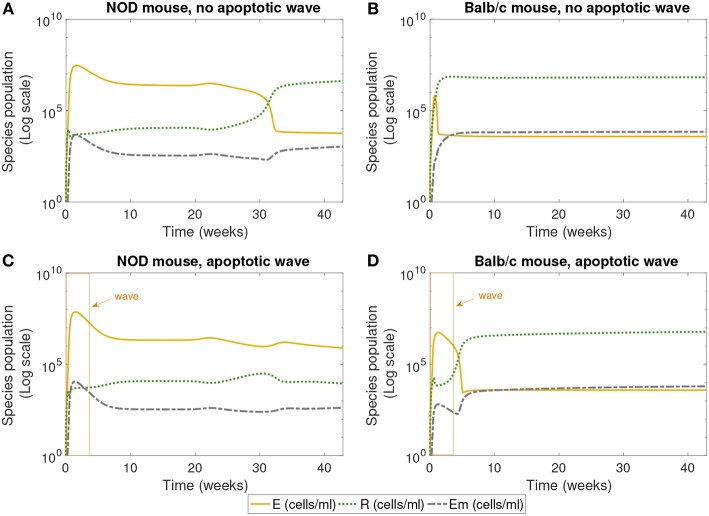
The populations of effector (*E*), regulatory (*R*), and memory (*Em*) T cells simulated for 40 weeks, according to the single compartment mathematical model. We compared the system in the presence and absence of an early wave of apoptosis, and between NOD and Balb/c mice. The NOD mouse shows elevated effector T cells for large values of time in the presence of the apoptotic wave, an indicator of an autoimmune response, while the Balb/c mouse and the NOD without a wave have elevated regulatory T cells, a sign of immune homeostasis. **(A)** NOD mouse, no apoptotic wave, **(B)** Balb/c mouse, no apoptotic wave, **(C)** NOD mouse, apoptotic wave, and **(D)** Balb/c mouse, apoptotic wave.

**Figure 4:**
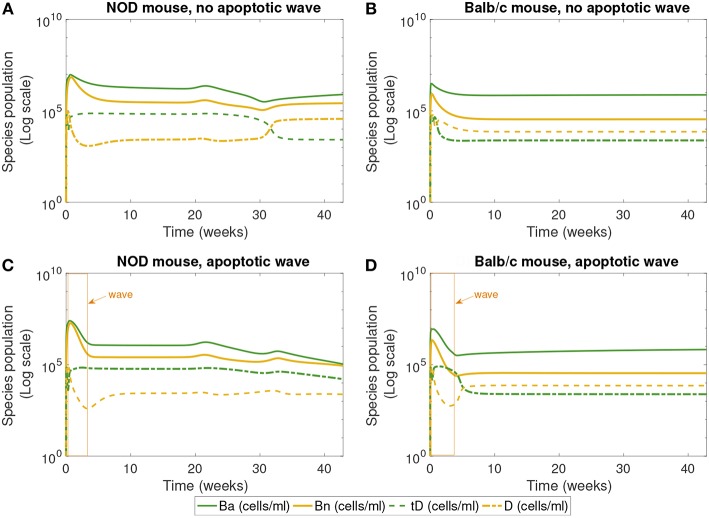
The populations of apoptotic β-cells (*B*_*a*_), necrotic β-cells (*B*_*n*_), tolerogenic DCs (*tD*), and immunogenic DCs (*D*), simulated for 40 weeks, according to the single compartment mathematical model. We compare the system in the presence and absence of an early wave of apoptosis, and between NOD and Balb/c mice. **(A,C)** Show that the NOD mouse exhibits higher levels of necrotic β-cells, which are thought to contribute to the development of T1D. In **(C)**, the NOD mouse in the presence of an apoptotic wave shows decreased apoptotic β-cells. **(A)** NOD mouse, no apoptotic wave, **(B)** Balb/c mouse, no apoptotic wave, **(C)** NOD mouse, apoptotic wave, and **(D)** Balb/c mouse, apoptotic wave.

One aim of previous model-based studies of T1D was to evaluate possible triggers of T1D (Marée et al., 2006). Similarly, we first simulated the model in the presence and absence of one proposed trigger, an early period of β-cell death in the pancreas (i.e., apoptotic wave). Additionally, the model was simulated for two parameter sets, one based on data from healthy Balb/c mice, and the other based on data from NOD mice that develop T1D. The results of these simulations are present in Figures 2–4.

The dynamics of healthy β-cells, glucose, and insulin are illustrated in Figure 2. These populations most clearly indicate whether or not the system enters a diseased state. In our simulations, we have set the threshold for pathologically elevated levels of glucose generated in our model to be 250 mg/dl, a choice motivated by Li and Escher (2013). Recall that in neonatal rodents (both NOD and healthy Balb/c mice), the pancreas undergoes structural changes due to a high rate of apoptosis of β-cells (the apoptotic wave) during weaning (Trudeau et al., 2000). We approximated the action of an apoptotic wave in our model through function *W* (where *W*(*B, t*) ≥ 0.01*B*). We indicate the effective range of our simulated apoptotic wave by a box in Figures 2C,D. In these model simulations, in the absence of an apoptotic wave, neither the NOD mouse nor the Balb/c mouse develops pathologically high glucose levels. However, even without the apoptotic wave, the NOD mouse does experience a period of β-cell death in the first 30 weeks of the simulation.

In the simulation, when the apoptotic wave is introduced, the NOD mouse develops clinically elevated glucose, along with depleted levels of β-cells and insulin, but the simulated Balb/c mouse does not. Simulated NOD glucose levels cross the 250 mg/dl threshold at approximately 24 weeks. On the other hand, after the wave, the Balb/c mouse is able to “recover” while the β-cell mass in the NOD mouse continues to decline. The simulation was run for approximately 40 weeks which aligns with time scales of experimental observations. The simulations show that the numerical steady state glucose value for the healthy systems is approximately 89 mg/dl (which is comparable to a healthy value of 100 mg/dl computed in Topp et al., 2000), while the simulated steady state glucose level for the diseased system *in silico* is approximately 539 mg/dl, well above the diagnostic level.

Next we examined the dynamics of specific immune cells in this simulation, shown in Figure 3. Figure 3A shows that in the case when there is no apoptotic wave, the NOD mouse nonetheless has a period of elevated effector T cells, corresponding to the period of decreased β-cell mass observed above. However, as time progresses, the concentration of regulatory T cells increases, and β-cell mass recovers, thus avoiding the development of T1D. The *in silico* NOD mouse shows higher levels of effector T cells when compared with the Balb/c mouse. In the case of the Balb/c mouse without a wave, we can see that effector T cells decrease early on, and regulatory T cells increase, with little impact on the population of healthy β-cells.

When we add the simulated apoptotic wave, both systems display an initial spike in effector T cells. After the period of the apoptotic wave, regulatory T cells increase in the Balb/c mouse. For the NOD mouse, there is a decrease in effector T cells after the apoptotic wave, but they remain elevated, corresponding to the long-term depletion of β-cells. In the simulation results shown in Figure 3, regulatory T cells do increase after the wave in NOD mice, but do not reach sufficient levels to inhibit the effects of effector T cells. All systems display similar levels of memory T cells, although they are slightly higher in the Balb/c mouse.

Finally, Figure 4 shows the populations of apoptotic and necrotic β-cells and immunogenic and tolerogenic DCs. (Note that active and inactive macrophages were tracked for this simulation, but the results are not plotted. Both the macrophage populations quickly reach a steady state value and remain there, and their levels do not vary greatly between NOD and Balb/c mice.)

The first 30 weeks of model simulation show some complicated nonlinear interactions among the immune cell populations. When there is no apoptotic wave (Figures 4A,B), the NOD system displays slightly higher levels of necrotic β-cells than the Balb/c mouse due to differences in macrophage clearance rates between NOD and healthy mice. Immunogenic DCs in NOD mice start at a high level due to the presence of higher levels of necrotic cells. These necrotic cells in turn produce an upregulation of effector T cell levels. The effector T cell increase is followed by rapid depletion of DCs via negative feedback arising from the direct removal of DCs by effector T cells in our model. On the other hand, in the absence of the apoptotic wave, the apoptotic cell levels are not sufficient to elicit high levels of tDC in NOD mice. These lower levels of tDC in NOD mice lead to lower levels of regulatory T cells, which then regenerate higher levels of tDC up to week 30. After this initial time frame, there is a tDC induced boost in regulatory T cells followed by a decline in tDC levels through negative feedback in NOD mice.

Although we observe that, without the apoptotic wave, the NOD system shows initial nontrivial dynamics in DC vs. tDC levels, the system eventually returns to a healthy state. In contrast, the Balb/c mouse without the apoptotic wave moves directly into a healthy state, since macrophage clearance rates are sufficiently high to remove necrotic cells.

In the presence of the apoptotic wave in Figures 4C,D, both simulated systems show a larger initial spike in apoptotic β-cells. As apoptotic and necrotic β-cells increase, and healthy β-cell mass decreases, it leads to a dip in immunogenic DCs in both types of mouse due to a fast increase in effector T cells. However, in the Balb/c mouse these effects are temporary, while in the case of the NOD mouse the immunogenic DCs remain at a lower level. Tolerogenic DCs are elevated in the sick NOD mouse relative to the Balb/c mouse, since there are more regulatory T cells at steady state in Balb/c than in NOD mice in Figure 3. The NOD mouse has higher steady state levels of necrotic β-cells than Balb/c after the apoptotic wave, which indicates a higher state of inflammation. Yet, as more immunogenic DCs are being produced in response to abundant NOD necrotic β-cells, they also become quickly eliminated by high levels effector T cells.

The levels of DC and tDC populations in the NOD vs. Balb/c mice may at first appear to be counter-intuitive: the NOD mice that have achieved a diabetic state (Figure 3C) have higher levels of tDCs than DCs, as compared to the healthy Balb/c mice that have higher DC levels than tDC levels. This dendritic cell response is in contrast to the steady state response of the T cells we see in Figure 3. Simulations produce high effector T cell levels in diabetic NOD mice and high regulatory T cell levels in healthy mice, which is consistent with our intuition that high inflammatory signals correspond to a diseased state. The nonlinear feedback dynamics of our model yield this inverse relationship between steady state levels of tDCs and regulatory T cells, and between DCs and effector T. This is because in our model, the dendritic cell populations increase or decrease in response to the presence or absence of inflammatory signals, whereas the regulatory and effector T cell populations increase or decrease in response to the tDC and DC signals. Thus, once the anti-inflammatory regulatory T cell levels are sufficiently high, for example, the tDC population is no longer needed, and will be reduced. Similar dynamics take place between DCs and effector T cells with the inflammatory signals.

**Figure 5:**
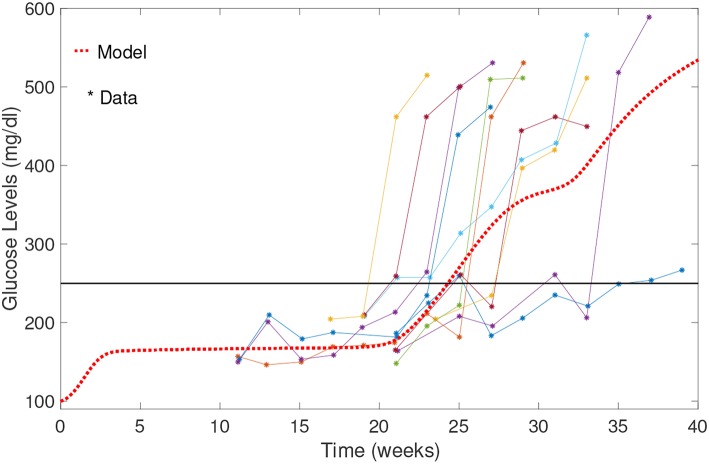
Comparison of NOD model results with data from Li et al. (2010). Model results capture the average biological system response. Note the initial increase in blood glucose levels; glucose levels stabilize between weeks 3 and 20, and then rise relatively rapidly to above the 250 mg/dl threshold.

A comparison of our model results against data from Li et al. (2010) is shown in Figure 5. Our model can capture average glucose dynamics well; however, there is substantial variability in glucose dynamics between individual mice, particularly as related to time of diabetes onset. In the simulated glucose dynamics, we note an initial glucose increase that is followed by a leveling out for a period of time, followed by a subsequent increase. From week 3 to week 20, β-cell destruction is taking place due to immune engagement in the pancreas. Nevertheless, symptoms are not apparent until after week 20. The rapid increase in glucose levels after week 20 in our model seems to follow an increase in effector T cell effectiveness against β-cells.

### Sensitivity Analysis

**Figure 6:**
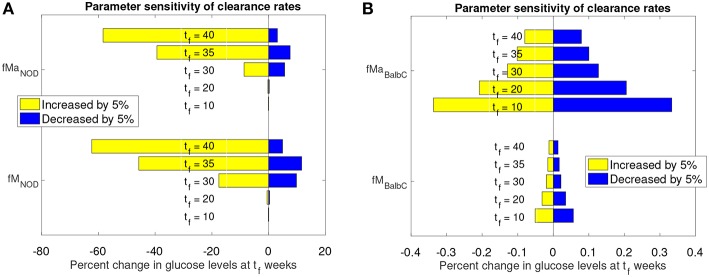
Sensitivity analysis of clearance rates *f*_*M*_ and *f*_*Ma*_ for NOD and Balb/c mouse models. **(A)** Effect of 5% clearance rate changes for the NOD mouse on glucose levels at different weeks, *t*_*f*_. **(B)** Effect of 5% clearance rate changes for the Balb/c mouse on glucose levels at different weeks, *t*_*f*_.

We numerically explored the sensitivity of each of the model parameters one-at-a-time. Each parameter was both increased and decreased individually while the remaining parameters were fixed. Since the macrophage clearance rates *f*_*M*_ and *f*_*Ma*_ are the main factors in distinguishing the fate of the NOD from the Balb/c mice, we present an analysis of the impact of changing those clearance rates in Figure 6.

The graphs in Figure 6 show the percent change in simulated model glucose levels at *t*_*f*_ weeks. The color of each bar represents whether the parameter was increased or decreased. Simulations for both the NOD and Balb/c mice in this case include the apoptotic wave. This ensures that, in the absence of any parameter modifications, the NOD mice will become diabetic in our model.

For the NOD mouse (Figure 6A), we see that during the transient period (up to week 35), the sensitivity of the model outcome to a 5% change in the clearance rate parameter values increases over time. A 5% increase in the clearance rates leads to approximately 60% reduction in glucose levels by week 40. The response to a decrease in the clearance rates is not symmetric: a 5% decrease leads to less than a 20% increase in glucose levels.

Compared to NOD mice, Balb/c mice (Figure 6B) show very little sensitivity to clearance rate changes over the same time period (note the *x*-axis value differences in Figure 6A vs. Figure 6B). For the non-diabetes-prone Balb/c mice, the glucose levels change by <1% at any time point, and respond fairly symmetrically to an increase or decrease in clearance rates. In fact, the Balb/c model was fairly insensitive, not only to the clearance rates, but to any parameter changes. We observed (data not shown) that for the Balb/c mouse, when parameters were changed by 5% from their original value, there was virtually no impact on steady state glucose levels.

**Figure 7:**
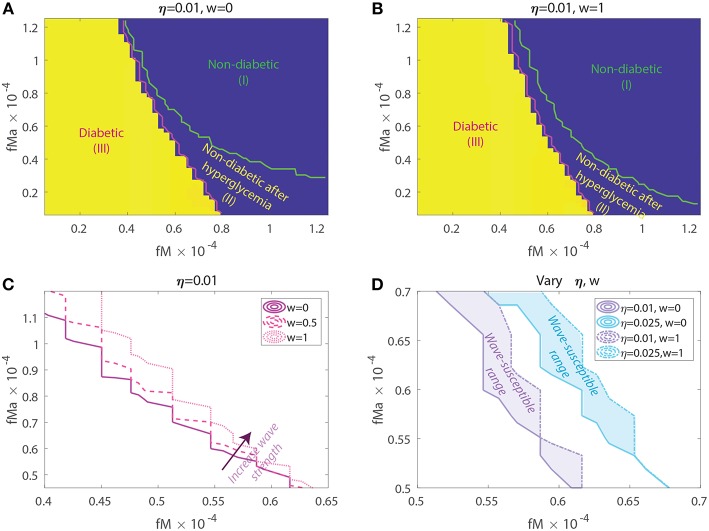
Susceptibility to acquiring diabetes as a function of apoptotic wave strengths *w*, macrophage clearance rates *f*_*M*_ and *f*_*Ma*_, and of baseline T-effector activity, η. **(A)** No apoptotic wave: Heat map of model glucose values at 142.85 weeks for varying macrophage clearance rate combinations without apoptotic wave. **(B)** With apoptotic wave: Heat map of model glucose values at 142.85 weeks for varying macrophage clearance rate combinations. **(C)** Changes in healthy boundary between region (III) and (II) as wave scaling, *w*, is increased with fixed η = 0.01. **(D)** Clearance rate regions that transition from a healthy to a diseased state with the addition of the wave (wave susceptibility range in shaded regions). Two susceptibility regions are computed, one for η = 0.01 and one for η = 0.025.

Motivated by the above sensitivity results for model parameters, we explored the model response for a few key parameter combinations that are critical to the mechanism for disease development in our model. For this purpose, we used a Latin Hypercube sampling (LHS) scheme to generate pairwise combinations of macrophage clearance rates, *f*_*M*_, *f*_*Ma*_. Briefly, LHS is a stratified Monte Carlo sampling scheme that assigns a distribution for each parameter, partitioning them into N subintervals of equal probability then independently sampling without replacement from each subinterval. We sampled uniformly from the following clearance rate intervals $f_{Ma}\in \left[0.062,1.2\right]\times 10^{-4}$ and $f_{M}\in \left[0.062,3.1\right]\times 10^{-4}$ with interval upper bounds corresponding to the Balb/c set of clearance rates. For each interval we chose 50 subintervals for sampling corresponding to a total of 2,500 parameter combinations. In addition, in order to test the model response as a function of the wave, and the basal effector T cell activity, η, the LHS clearance rate combinations were used in order to simulate the model for three wave scales (*w* = 0, *w* = 0.5, *w* = 1) and two η = 0.01, 0.025. Representative model results from these simulations are shown in Figure 7.

**Figure 8:**
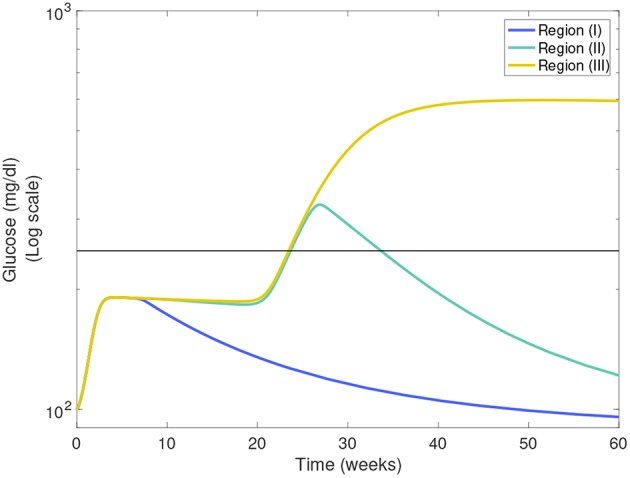
Representative glucose levels for the three clearance regions from Figure 7B: (I) non-diabetic, (II) non-diabetic after hyperglycemia, and (III) diabetic.

In Figures 7A,B we show heat-maps of long term model glucose levels at 142.85 weeks (1,000 days) against the macrophage clearance rates either without an apoptotic wave Figure 7A or with apoptotic wave Figure 7B. Three regions emerge in our simulations: (I) high macrophage clearance rate combinations that produce non-diabetic mice at the end of the simulations, (II) intermediate clearance rate combinations that produce non-diabetic mice that had a hyperglycemic episode at some point, (III) low clearance rate region in which the mice end up with high glucose levels. Three representative simulations of glucose levels with clearance rate parameters selected from each region are shown in Figure 8.

The boundaries between the three regions are marked in Figures 7A,B and their location can change as η and w vary. Of note here is that the boundary between region (II) and (III) (we refer to this as the healthy boundary) which shifts to the right as the magnitude of the wave is increased, as illustrated in Figure 7C. This indicates that the diabetic region (III) expands as the wave is added, engulfing clearance rate combinations that produced non-diabetic outcomes without the wave. This shows that there is a range of clearance rate combinations that are susceptible to the wave (ranges shown in Figure 7D). These susceptible ranges are of interest to us as they generate the NOD phenotype in our model, meaning that for these parameters the apoptotic wave shifts the system from the healthy to the unhealthy state. Indeed, for these parameters our model satisfies the Copenhagen hypothesis. In addition to the wave, we also observe that as the effector T cell activity (η) is increased the wave-susceptible clearance rates ranges shift (Figure 7D) indicating that the NOD phenotype can be observed with higher macrophage clearance rates if effector T cells are more efficient at targeting β-cells.

In addition to the healthy boundary transitions, there are three additional important ideas that emerge from studying macrophage clearance rate combinations. First, we note that the diabetic region (III) includes regions where *f*_*Ma*_ > *f*_*M*_ indicating that out of the two clearance rates, the inactivated macrophage clearance rate seems to transition the system into a diabetic state when lowered significantly despite *f*_*Ma*_ being larger. Second, in Figure 7B we note that region (II) might be of biological significance; note that this region is one in which mice have lower clearance rates than the Balb/c range (recall that $f_{M}\in 1.2\times 10^{-4},f_{Ma}\in 3.1\times 10^{-4}$), yet they are able to recover from hyperglycemic episodes in the presence of the apoptotic wave. This subset of clearance rates, could be of relevance in the context of NOR mice as in O'Brien et al. (2002) it was observed that macrophages from female NOR mice were deficient in phagocytosis of apoptotic thymocytes compared with Balb/c macrophages, however their phagocytic ability was greater than NOD macrophages. Finally, we note that the Balb/c clearance rates used in the mathematical model are far away from the healthy boundary whereas NOD clearance rates are close to the healthy boundary; this could explain why the healthy Balb/c mice show tremendous robustness in the face of parameter perturbations whereas NOD mice were far more susceptible to parameter variations, in agreement with results in Figure 6.

### Simulating Potential DC Treatments for the NOD Mouse

Treatments that use injections of specifically targeted tolerance inducing dendritic cells have shown promise in recent T1D pre-clinical and clinical trials, reviewed in Creusot et al. (2014). Typically, vaccine dose is determined empirically in pre-clinical studies (Creusot et al., 2008, 2010, 2014; Ruffner and Robbins, 2010; Giannoukakis et al., 2011). In NOD mice the dosing of DC therapy ranges between 1 × 10^5^ and 5 × 10^6^ cells administered either once or by several injections spread out in weekly intervals. In this section, we investigate how our model responds to simulated DC treatments using current studies that have examined how the frequency and dose of injected tolerogenic DCs affects T1D disease progression. Specifically, we first closely examined two treatment plans corresponding to two total dose strengths, one representative low dose at 2 × 10^5^ and one representative high dose at 2 × 10^6^ using the ranges reported in NOD mice.

**Figure 9:**
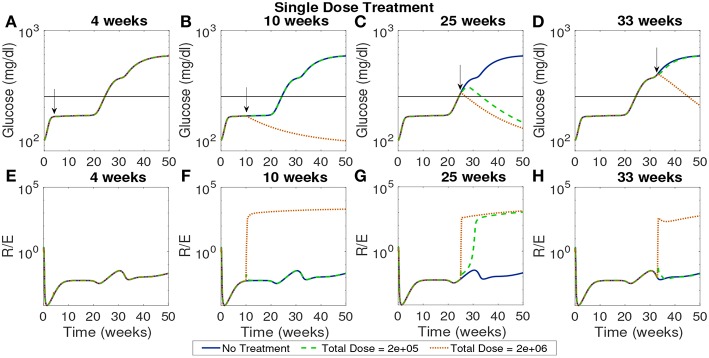
The NOD mouse model simulated with a single dose of tolerogenic DCs administered in a single injection, for varying dose amounts and treatment start times. **(A–D)** Glucose levels for all treatment levels. **(E–H)** Ratio of regulatory to effector T cells for all treatment levels. **(A)** Treatment is ineffective when started too early for any dose amount. In all cases, T cell ratios indicate larger values of effectors to regulatory cells when treatment is not effective. **(B)** Only high doses are effective when started at the 10 week mark in the pre-diabetic range. **(C)** Both treatment amounts are effective right after glucose threshold is exceeded, as indicated by low terminal glucose levels and high T regulatory cell levels. **(D)** Treatment can be effective when started after the development of the disease, but larger doses are required.

In Figure 9 we plot the model simulated with two single doses injected at various time points on a modeled NOD mouse system. Experiments have shown that the timing of injections can impact the effectiveness of treatment (Creusot et al., 2008, 2010; Ruffner and Robbins, 2010; Giannoukakis et al., 2011). Accordingly, we timed the injections in order to capture disease dynamics at important disease development points. Studies involving NOD mice have found that in mice that develop the disease, β-cell autoantibodies are first detected between 8 and 10 weeks, but that the development of an autoimmune response begins around 4 weeks (Creusot et al., 2014). We marked a cutoff of 250 mg/dl as the criterion for entering a full diabetic state, which is reached at 24.3 weeks of age for our modeled NOD mouse. Motivated by these points in disease development, we simulated four interesting cases of treatment start times: 4 weeks which is around the time the immune response is thought to be initiated, 10 weeks, or the time that autoantibodies are first detected, 25 weeks which is the time that this model is considered to develop the disease, and 33 weeks at which point the glucose levels experience a small plateau after the disease has progressed in our model.

In Figure 9 glucose and T cell ratios are shown for up to 50 weeks. Note that glucose levels above 250 mg/dl are considered evidence of the disease, and below 250 mg/dl, considered not to be overtly diabetic (marked by a horizontal line in the plots). In the cases in which glucose exceeds the diabetic threshold, we also see that effector cell levels exceed regulatory T cell levels. The cases in which the mouse shows non-diabetic glucose levels are also marked by higher levels of regulatory to effector cells, indicating an important role of T cells in disease development. The time at which treatment is applied can significantly affect outcomes. If the treatment is applied at the earliest time, 4 weeks, it does not prevent the development of disease, regardless of the dosage. At the 10 week mark at which point the model is in pre-diabetic stage, only the high dose is effective, whereas at 25 weeks both dosages are effective when administered in a single injection. At 33 weeks, only the higher dosage of 2 × 10^6^ cells has an impact, indicating that the treatment is less effective if given too long after the disease develops. Thus, a simple investigation of the model yields non-trivial time and dose dependency for DC injections at various stages of T1D development.

**Figure 10:**
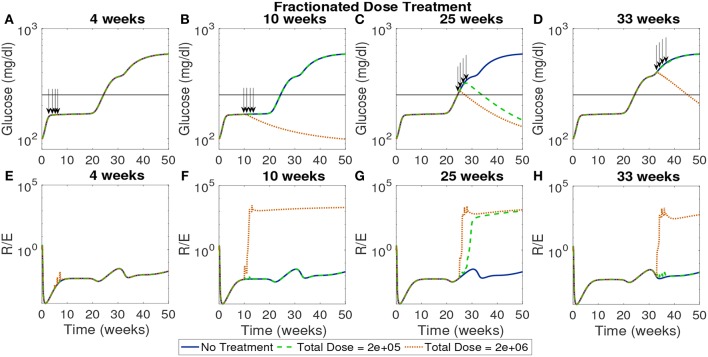
The NOD mouse model simulated with doses of tolerogenic DCs administered in four weekly injections, for varying dose amounts and treatment start times. **(A–D)** Glucose levels for all treatment levels. **(E–H)** Ratio of regulatory to effector T cells for all treatment levels. **(A)** Early treatment is not effective when it is delivered in four weekly doses for both high and low dosing. **(B)** Only high doses are effective and in **(C)** we see that for later treatment start times both low and high doses are effective, however, in **(D)** we see that the low dose loses effectiveness similar to the case with one dose application.

In Figure 10 the total dose levels remain the same as before, but each dose is fractionated in weekly installments across 4 weeks. In this case, with a start time of 4 weeks, all doses remain ineffective. This indicates that even when early intervention is distributed over a longer time span it cannot reverse the course of the disease. The rest of the treatment start times show similar response to the single dose examined earlier with the high dose showing effectiveness if initiated after the 10 week mark.

**Figure 11:**
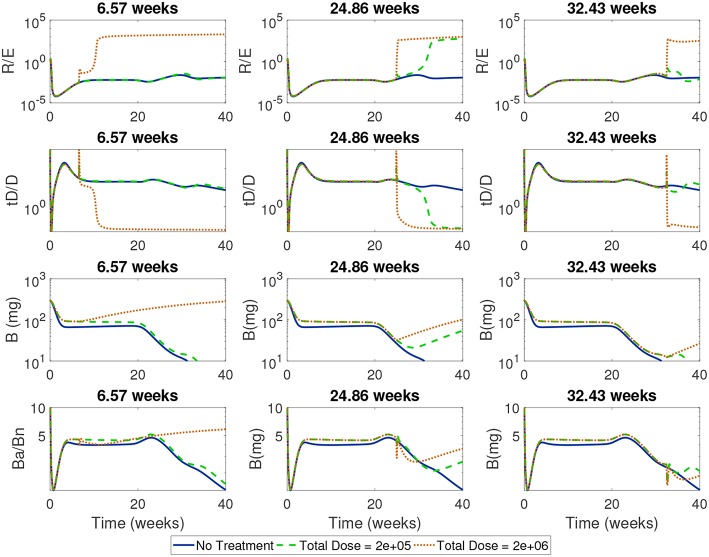
Time-course of cell ratios with two doses (2*e* + 05 and 2*e* + 06) with treatment started at critical transition start times. At Start Time = 6.57 weeks, the high dose first becomes effective displaying a rapid increase in regulatory T cells followed by a slow increase in β-cells; the low dose treatment, however, is not able to alter the course of T1D development. At Start Time = 24.86 weeks the low dose first becomes effective at eliciting a Treg response which corresponds to a switch in the ratio of *B*_*a*_/*B*_*n*_ and an increase in β-cell mass. At Start Time = 32.43 weeks the low dose is not effective anymore.

Next, we simulated treatments using a wide range of start times (from 1 day to 45 weeks) for the high and low doses in order to identify critical times when each dose first became effective or lost effectiveness. In Figure 11 we show the time course of the ratios of Treg to Teff, tolerogenic to immunogenic DC, and apoptotic β to necrotic β-cells around one critical transition point for the high dose, corresponding to 6.57 weeks, and two critical transition points for the low dose at 24.86 weeks (when low dose first becomes effective) and 32.43 weeks (when low dose is first ineffective).

For all the critical start times examined in Figure 11, we observed that the ratios of non-inflammatory to inflammatory cells took a dramatic turn if enough tDCs were injected at a particular time in the NOD mouse. Specifically, at the critical start times, tDC injections led to increases in regulatory T cell levels compared to effector T cells. This was then followed by an eventual decline in tDC levels compared to DC levels, a dynamic we attribute to negative feedback between T cells and dendritic cells in our model. (Note that this response is the same as the one we saw in the model without treatment: tDCs initiate an immune response but are eventually removed by regulatory T cells.) In addition, these changes were accompanied by an increase in β-cell levels in the pancreas and recovery of the mouse. Of particular interest are the ratios of regulatory to effector T cells which show non-monotonic response as a function of time in the cases when the treatment is either not effective or not applied.

In order to investigate more closely the time vs. dose dynamics, we next simulated the model with a single injection and varied the treatment start time one day at a time over the course of 45 weeks for a wide range of possible doses between 1 × 10^4^ and 5 × 10^6^ in dose increments of 10^4^. For these simulations, treatment outcomes were evaluated at 85 weeks in order to allow the system to settle into a steady state.

**Figure 12:**
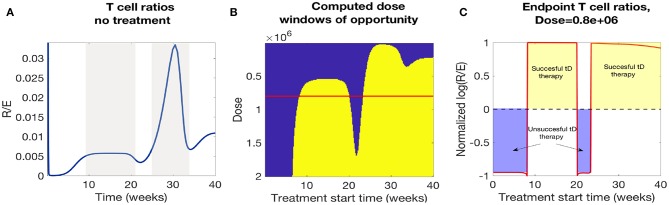
A comparison of NOD mouse T cell ratios with computed treatment windows. **(A)** An untreated NOD mouse time course of the ratio of regulatory (*R*) to effector (*E*) T cells. After the apoptotic wave an increase in regulatory cells due to cell death generates a peak in the ratio (*R*/*E*), which qualitatively agrees with the peaks in the low dose window of opportunity calculations. **(B)** The NOD mouse model simulated with a single dose of tolerogenic DCs administered at a series of treatment start times. Lighter regions indicate the treatment levels that successfully return the mouse to a healthy state. Note that dose levels decrease along the y-axis, so that higher doses are in the lower part of the graph, and lower doses are in the top part. Windows of opportunity change as a function of start time and tDC injection dose. **(C)** Windows of opportunity (in time) for an example tDC dose level (0.8e+06), comparing outcomes for treatment started at different times. The log ratio of *R*/*E* is plotted as a function of treatment start time: log (*R*/*E*) > 0 indicates treatment was successful, and log (*R*/*E*) < 0 indicates treatment was not successful.

In Figure 12A we plotted the time course of regulatory to effector cell ratios for an untreated NOD mouse along with effective dose time windows for all single tDC treatment doses tested in Figure 12B. First, for the untreated mice, we note that the ratio of regulatory to effector cells declines significantly following the apoptotic wave (Time< 10 weeks), as an inflammatory response is mounted in response to production of uncleared necrotic β-cells. However, after the initial post-apoptotic dip, regulatory cells recover and peak between week 10 and 20 followed by a secondary peak in regulatory T cells around week 30 (both peaks are marked in gray in Figure 12A). Upon comparison, we noted a striking similarity between the dose time windows and the untreated NOD T cell ratios (compare the peaks in Figures 12A,B). Specifically, windows of opportunity for treatment seem to correspond to the two major regulatory T cell peaks that occur in untreated mice. The increase in regulatory cells in these two cases seems to come right after significant β-cell death events, such as apoptotic wave, or when rate of effector T-cell strength (η_*e*_(*t*)) increases most rapidly [i.e., after the half-max point for η_*e*_(*t*)]. This indicates that windows of opportunity emerge when regulatory T cells experience slight increases following β-cell apoptosis. In Figure 12C, we illustrate the critical role of T cell ratios for a chosen dose (note red line in Figure 12B marks Dose = 0.8e6) where positive treatment outcomes correspond to simulations for which the steady-state *R*/*E* ratio is positive in logarithmic scale whereas negative treatment outcomes correspond to negative values in the logarithmic scale (compare Figures 12B,C).

In Figure 12B we observe roughly three dosing categories: (1) Low dose treatments (toward the top of Figure 12B) have a single late window of opportunity that decreases as the dose decreases and then disappears at 3*e*4, (2) Medium dose treatments that show two distinct windows of opportunity (early and late windows), (3) High doses (toward the bottom Figure 12B) show longer uninterrupted windows of opportunity that start at earlier times for increasing doses.

In conclusion, we see that there is a striking correspondence for the treatment effectiveness times for single tDC injections in our model with the periods of time for which the regulatory to effector cell ratios stay above a certain threshold. Based on this observation, we propose the following hypothesis: the time windows in which regulatory T cells seem to recover might be the most opportune times to administer tDC injections, as this may lead to subsequent disease recovery. This indicates that the (*R*/*E*) ratio might need to reach a particular threshold value before treatment can be effective and the specific threshold varies as doses vary.

## 4. Discussion

In this paper, we have presented a simplified single compartment mathematical model that incorporates dendritic cells and T cells interacting with pancreatic β-cells. This model captures important qualitative features of T1D progression and dynamics. First, the model has clear, distinct, steady states that match the dynamics observed in both healthy Balb/c and disease-prone NOD mice. Comparing simulation outcomes for the NOD or Balb/c systems, a mouse enters a diseased steady state only if it has both reduced efficacy of macrophages (typically associated with NOD mice) and undergoes a wave of increased β-cell death. Both of these characteristics have been observed in NOD mice, and implicated in their susceptibility to T1D in the context of the Copenhagen hypothesis (Maree et al., 2005). Importantly, we also have incorporated an active role of DCs in both clearance of dying β-cells and generation of a T cell response due to a direct β-cell death interaction in the pancreas, which, to our knowledge, has not been studied in detail in prior models. Our DC clearance rates were carefully fitted to published data and work in conjunction with macrophages to create either a healthy or a diseased state in our model. Our model included multiple immune pathways and nonlinear feedback dynamics. For example, we observed that tolerogenic DCs are needed in order to initiate a regulatory T cell response, which is necessary for mice to reach a healthy state. Counter-intuitively, however, simulations of a healthy NOD mouse result in low levels of tolerogenic DCs during the maintenance of a low inflammatory state, in which effector T cell levels are high, and the disease does not prevail. This is a model prediction that needs to be validated through biological experiments.

While our model only includes the pancreatic compartment, we necessarily have to include many parameters that capture the dynamics of the interactions between immune cells and β-cells. Many of these parameters have been measured or inferred, and we carried out our own fitting where possible as well. Yet, model response must be tested for sensitivity to parameter values. From our sensitivity analysis, we see that the NOD mouse is much more sensitive to single parameter changes than is the Balb/c mouse. We interpret this to mean that the healthy clearance rates of the simulated Balb/c mouse create a more robust healthy system overall, so that no single factor can easily lead to disease development. Interestingly, a number of parameter modifications in the NOD case will drive the system toward a healthy steady state. This supports a scenario in which the model has two distinct steady states, corresponding to healthy and diseased physiological conditions, and that within the range of parameters examined, the “diseased” state has a smaller basin of attraction. Since the model is currently highly non-linear, a steady state analysis is a challenge we aim to explore in the future. Increases in either the active or inactive macrophage clearance rates can protect the NOD mouse from developing the disease, further supporting the hypothesis that macrophage clearance plays a role in the development of T1D. In addition, by studying a large set of clearance rate combinations, we discovered non-trivial relationships between macrophage clearance rates, apoptotic wave magnitude, and effector T cell kill rates for β-cells. These observations might give us some clues as to why we see large variability in individual mouse glucose level time courses for the NOD mouse. We intend to explore population variability further in the future.

The results of the model simulations are encouraging in the context of developing simulations for a DC-based treatment for T1D. We note that our model is significantly simplified by virtue of allowing all interactions to take place in the pancreas. Nonetheless, the responses of the model to external infusions of tolerogenic DCs supports the concept that treatments targeting the tDC population are worth investigating further, both biologically and through expanded mathematical models. In our model the balance of effector and regulatory T cells depends on the balance between apoptotic and necrotic β-cells, and thus on the ratio of DCs and tDCs. Changing that balance of DC levels can have an impact on disease progression. We thus accordingly tested clinically relevant tDC treatments and observed that both timing and dosing of tDC administration can have a significant impact on the ability of the NOD mouse to resist disease onset and to recover. Most interestingly, in agreement with data from Feili-Hariri et al. (2003), Creusot et al. (2008, 2014), and Ruffner and Robbins (2010), we observed that treatment was most effective if it was applied later rather than earlier in the NOD mouse, but if applied too late it could not affect disease outcome. High doses were the most effective in our model, but even those could not lead to a healthy state before some immune involvement was present in the NOD mouse. This may indicate that preventative treatments might not be as effective in this context. These observations may support the concept that a certain level of inflammation may be required for DC-based treatments to be effective. An observable change in the direction of disease progression might also be more apparent when β-cell loss or dysfunction is near a certain threshold below which blood glucose levels continue rising. Assuming that β-cells can divide if protected from autoimmune destruction, timing of treatment may result in β-cell counts rising back to the threshold that allows the subject to resume insulin secretion at normal levels thus restoring normal blood glucose levels. If β-cell counts are at the threshold level or far from it, then a bigger change is required to observe noticeable change.

In preclinical studies, it is very difficult to explore outcomes for a large range of doses and timings, but with a mathematical model such as this one, we are able to investigate a much wider range of treatment protocols. Such an exploration revealed interesting dynamics that were affected both by using various levels of dosing, and by starting the dosing at different times through the life of the *in silico* mouse. With simulated intermediate dose levels, unexpected windows of opportunity for treatment efficacy emerged as the system recovered post apoptotic wave: treatment that was applied early or late in the life of the simulated mouse was effective, yet there was also an interval of time in between in which intermediate dose levels were ineffective. Of particular interest is to understand how to predict when these time-windows of opportunity will arise. We discovered a strong connection between emergence of time-windows of opportunity for treatment initiation and higher ratios of regulatory to effector T cells in untreated NOD mice. This Treg to Teff ratio provides a nondimensional measure by which treatment effectiveness might be predicted prior to treatment. These results indicate that both dosing and treatment timing should be carefully examined in the context of T1D, particularly when immunotherapy treatments are under consideration.

The goal of this work was to develop a differential equations model of the biological systems involved in the development and treatment of type 1 diabetes, with an emphasis placed on the impact of DCs on the activity of T cells in the context of treatment. Models of this form are useful in understanding what triggers the development of T1D, how the disease progresses, and the potential impact of various treatments. There are multiple directions for future research using similar modeling techniques. First, the model could be expanded into a multiple organ compartment model that could more accurately capture organ-specific dynamics, such as immune interactions in the spleen or lymph nodes. This is an approach that we are currently pursuing. In addition, many immune cell interactions are complex and will require further data, such as data that can elucidate the impact of cell death on the tDC to DC ratio. Another aspect of the model to explore is the possible role of a delay in the production of T cells vis a vis modulation of the immune system by DCs. There is disagreement in the literature (Wodarz, 2007) over whether or not there is a waiting period when a T cell is activated by a DC. We chose to model the system without that delay, but it could provide additional insight or new and interesting behavior if we were to include it. Additional analysis is also possible for the simulation of DC therapy for T1D. While we examined treatment plans grounded in current experimental work, the model could be used to determine optimal treatments within this modeling framework. Variables to examine in the future include total dose, number of doses, time between doses, and treatment start time. We note that in this model, we focused on specific elements of T1D initiation and dynamics, so we chose not to incorporate β-cell viability as an additional factor. In future work, the inclusion of β-cell viability may also be important when exploring optimal treatments of the whole system. Finally, further comparison with experimental results may suggest modifications of the dynamics reflected in the model or treatment equations.

This report primarily provides three useful insights into modeling T1D. First are the apoptosis dependent activation for DCs and values of the β-cell engulfment rates by DCs in the pancreas. The values of the engulfment parameters themselves may prove useful in further attempts to refine the model dynamics of DCs in the pancreas. Additionally, the parameter fitting algorithm presented in this report can be expanded and improved. For example, the simplified differential equation model that we used to implement the parameter fitting algorithm does not agree with published data for long time periods (>2 h). While we mainly focused on transient behavior of DCs, this could indicate that there are additional dynamics to take into account in this simple set up. If this is the case, then further parameter fitting with a more detailed model of DC behavior could yield more accurate parameter values for use in modeling. Second, we provided a platform in which immune therapies can be examined, such as in the context of clinically relevant DC injections. This provides the ground work for more detailed multi-compartment models that examine immune cell activation in lymph nodes/spleen and their trafficking in the blood prior to engagement with the pancreas. We are currently pursuing these model extensions. And third, we discovered a strong connection between regulatory to effector T-cell levels and time-windows of opportunity that should be targeted for administering tDC treatments.

Finally, this model serves to continue building a framework for useful mathematical models of the development of T1D. The single compartment is unique because of the diversity of cell types that it incorporates into a model of T1D. However, other models, such as the one in Moore and Adler (2016) examine aspects of the system in more detail. Our model could serve as a baseline that could be extended to examine more detailed dynamics in a broader context. Additionally, it could be used to explore the impact of other outside factors on the development of T1D, such as different treatments, environmental factors, or physiological factors such as the presence of infection.

## Data Availability

The raw data supporting the conclusions of this manuscript will be made available by the authors, without undue reservation, to any qualified researcher.

## Author Contributions

LP and BS contributed conception, design, and numerical simulation of the study. MG contributed mathematical model modification and numerical simulations. AD contributed parameter sensitivity analysis. BS, MG, and LP wrote the first draft of the manuscript. SS and LE contributed support for study design, data, and participated in manuscript editing. BS and LP contributed writing, editing, and preparation of final manuscript draft. All authors contributed to manuscript revision, read, and approved the submitted version.

### Conflict of Interest Statement

SS and LE have financial interest in Aditx Therapeutics, a biotech company engaged in development of nucleic-acid-based products for induction of immune tolerance. The remaining authors declare that the research was conducted in the absence of any commercial or financial relationships that could be construed as a potential conflict of interest.
