# Supplementary material for: A Mathematical Model for DC Vaccine Treatment of Type I Diabetes
Source: Front Physiol. 2019 Sep 6;10:1107. doi: 10.3389/fphys.2019.01107 (PMC6742690; doi:10.3389/fphys.2019.01107)
Supplement: Supplementary file 1 [file Presentation_1.pdf]

Supplement to *A mathematical model for DC vaccine treatment of type I diabetes.*

Authors: Blerta Shtylla, Marissa Gee, An Do, Shahrokh Shabahang, Leif Eldevik, and Lisette dePillis

Maximum Likelihood Estimate Parameter Fitting

To fit the parameters in the simplified model, we used the Maximum Likelihood Estimate (MLE), implemented using the Metropolis Monte Carlo Markov-Chain (MCMC) algorithm for this process. In implementing MLE, we seek to find values of g_P that maximize the probability of obtaining the observed data, assuming the true values are represented by the parameterized model. We compute the difference between our model and the observed data using the χ^2 error, which is defined as follows: Let $D(t_i)$ represent the observed data, and $\rho(t)$ is the percent phagocytosis predicted by our model, then

$$\chi^2 = \sum_{i=1}^{N} \frac{(D(t_i) - \rho(t_i))^2}{\sigma_i^2}$$

where σ_i^2 is an estimate of the variance or error associated with each $D(t_i)$. Our algorithm calculates the likelihood that the data arose from the model using the likelihood function

$$P(D(t_i)|g_P) = e^{-\chi^2}$$

following the method outlined in [1, 2].

The implementation of this algorithm begins from an initial guess of the parameter values, and then selects new values by adding a value from the standard normal distribution scaled by our estimate of σ_i. In practice, we estimated σ_i based on the scale of the parameter being fit (note that χ^2 values in Figure 2 are not scaled by σ_i). We run the algorithm until successive values fall below a certain tolerance, which is determined based on the scale of the parameter values we are examining. At the end of a run, the final parameter value and the associated χ^2 are returned, and represent the parameter value selected by that run of the algorithm. Since we are using a stochastic algorithm, each time the algorithm is applied, we expect different results, so we validated the fitting results by comparing multiple trials of the fitting algorithm for each population of interest.

Results

Figure 1 displays the results of the selected parameters for tDCs, DCs, and macrophages. Qualitatively, the fit appears best for tDCS, where the trajectory of the model most closely matches experimental data. In the case of DCs, the low steady state value means that the model appears almost linear, because it must grow at a slow rate to match the experimental data. Thus, even though the curve is close to the data points, the overall shapes of the two sets are not similar. For macrophages the opposite problem occurs. Because the steady state value achieved by macrophages is higher than the possible steady state with this simplified model, the curve must increase very rapidly to minimize the distance to data points. In this case the simulated model ends up looking more curved than the experimental data.

Because we used a stochastic parameter fitting algorithm we expect some amount of variation between each run of the algorithm. In order to validate our use of the algorithm, we can verify that the results are consistent across multiple runs of the algorithm. Additionally, since we are attempting to solve a global optimization problem, this could show whether the algorithm is returning values

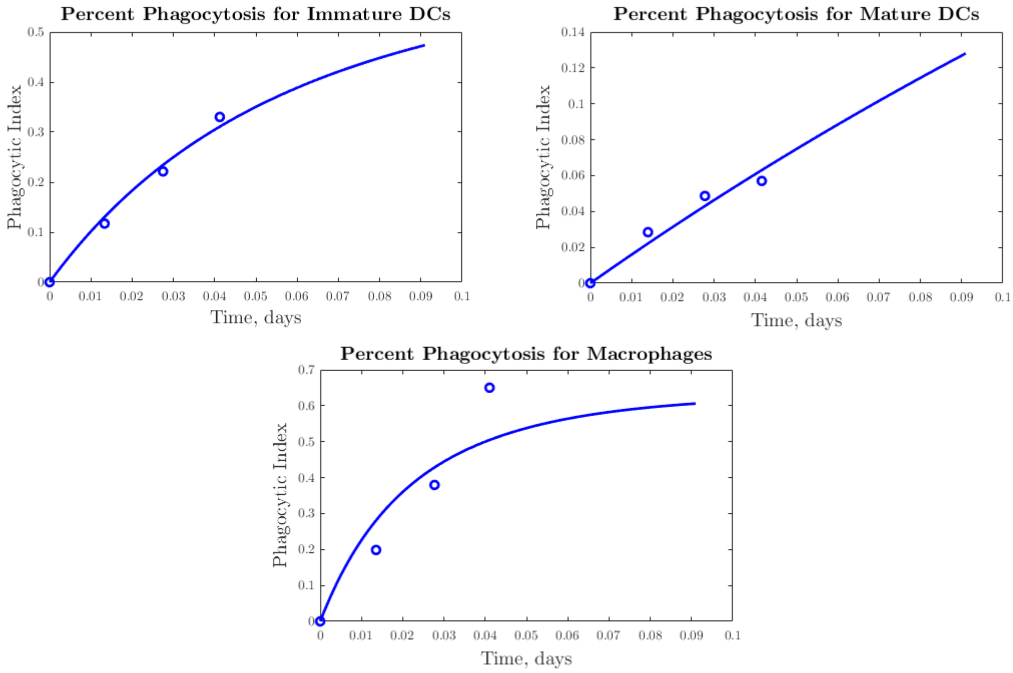

Figure 1: Simplified ODE model results against immature DC, mature DC and macrophage data from [3]. (A) The simplified ODE model plotted against data for immature DCs. This simulation is using $g_{tD} = 1.13 \times 10^{-5}$ ml cells^{-1} days^{-1}, selected from 15 runs of the MCMC fitting algorithm. (B) The simplified ODE model plotted against data for mature DCs. This simulation is using $g_D = 1.62 \times 10^{-6}$ ml cells^{-1} days^{-1}, selected parameter from 15 runs of the MCMC fitting algorithm. (C) The simplified ODE model plotted against data for macrophages. This simulation is using $g_{M_a} = 2.96 \times 10^{-5}$ ml cells^{-1} days^{-1}, the selected parameter from 15 runs of the MCMC fitting algorithm.

from multiple local minima of our χ^2 error, rather than the global minimum. Consistency across multiple runs supports the claim that our algorithm is locating a global minimum of our error function.

In order to evaluate performance across multiple runs of the data fitting algorithm, we ran the algorithm 15 times for each type of phagocytic cell. Figure 2 displays the results of these repeated trials for tDCs, DCs and macrophages. We see that parameter values appear narrowly distributed within a single range of values, so the multiple trials did not reveal multiple local minima in our model. In addition, the χ^2 values fell within a narrow range after each trial, indicating that the algorithm converged to a similar level of precision across multiple runs.

References

[1] Gregory P. Markov Chain Monte Carlo. In: Bayesian Logical Data Analysis for the Physical Sciences: a comparative approach with Mathematica support. 1st ed. Cambridge, UK: Cambridge University Press; 2010. p. 312–351.

[2] Chow C. MCMC and fitting models to data; 2010. Available from: https://sciencehouse.wordpress.com/2010/06/23/mcmc-and-fitting-models-to-data/.

[3] Albert ML, Pearce SFA, Francisco LM, Sauter B, Roy P, Silverstein RL, et al. Immature dendritic cells phagocytose apoptotic cells via $\alpha v\beta 5$ and CD36, and cross-present antigens to cytotoxic T lymphocytes. Journal of Experimental Medicine. 1998;188(7):1359–1368.

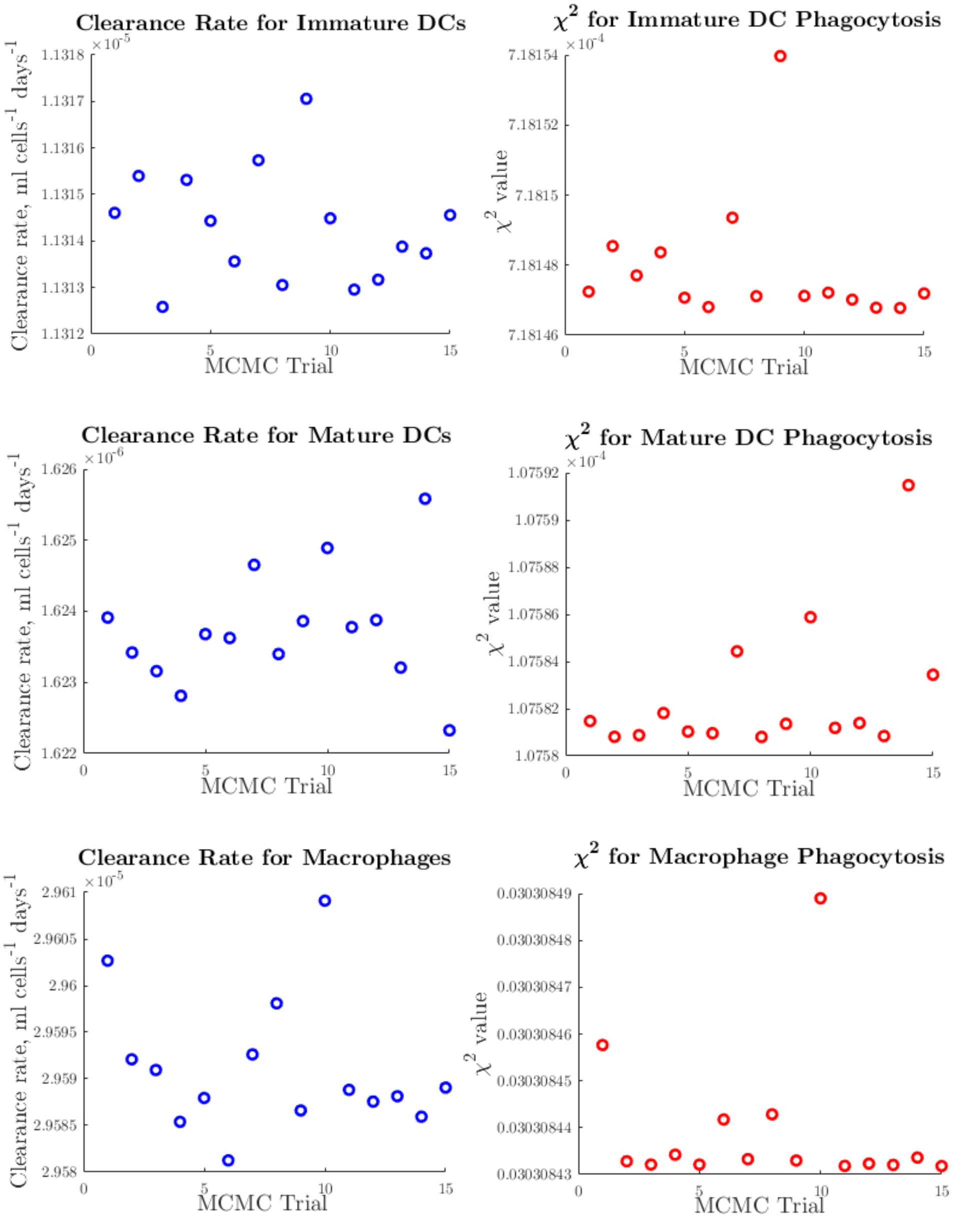

Figure 2: Results from 15 runs of MCMC data fitting algorithm for immature DCs, DCs and macrophages. (A) For tDCs the lowest values of the χ^2 error attained was 7.18×10^{-4}. The clearance rate associated with this value is $g_{tD} = 1.13 \times 10^{-5}$ ml cells^{-1} days^{-1}. (B) For DCs The minimum χ^2 error is given by 1.08×10^{-4}, with associated clearance rate $g_D = 1.62 \times 10^{-6}$ ml cells^{-1} days^{-1}. (C) For macrophages the minimum χ^2 is given by 0.030, with associated clearance rate $g_{M_a} = 2.96 \times 10^{-5}$ ml cells^{-1} days^{-1}.

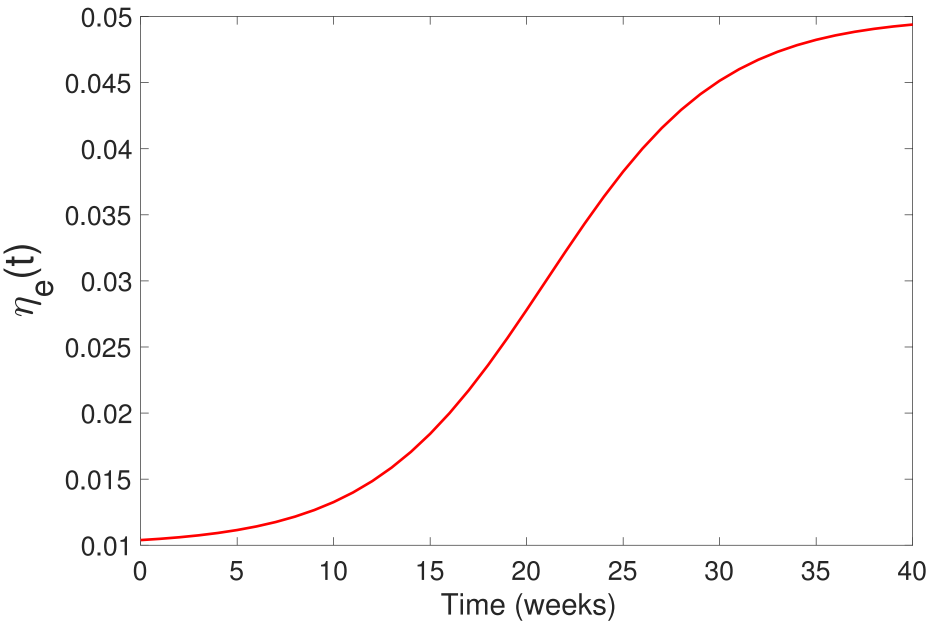

Figure 3: Plot of the effectiveness of β-cell killing by effector T cells, $\eta_e(t)$.
