# Supplementary material for: A Mathematical Model for DC Vaccine Treatment of Type I Diabetes
Source: Front Physiol. 2019 Sep 6;10:1107. doi: 10.3389/fphys.2019.01107 (PMC6742690; doi:10.3389/fphys.2019.01107)
Supplement: Supplementary file 2 [file Data_Sheet_1.pdf]

Appendix to *A mathematical model for DC vaccine treatment of type I diabetes.*

Authors: Blerta Shtylla, Marissa Gee, An Do, Shahrokh Shabahang, Leif Eldevik, and Lisette dePillis

Model Equations

$$\frac{d}{dt}M = J + (k+b)M_a - cM - f_M M B_a - f_M M B_n - e_1 M(M + M_a), \tag{1}$$

$$\frac{d}{dt}M_a = f_M M B_a + f_M M B_n - kM_a - e_2 M_a(M + M_a). \tag{2}$$

$$\frac{d}{dt}B = \alpha_B K_1(G)B - \delta_B B - \eta_e(t)K_2(E,R)B - W(B,t). \tag{3}$$

$$K_1(G, G_{hb}) = \frac{G^2}{G^2 + G_{hb}^2}. \tag{4}$$

$$K_2(E,R) = \frac{(s_E E)^2}{1 + (s_E E)^2 + (s_R R)^2}, \tag{5}$$

$$\eta_e(t) = \eta + 2\eta(1 + \tanh(\alpha_e(t - \beta_e))). \tag{6}$$

$$W(B,t) = .1wBe^{-\left(\frac{t-9}{9}\right)^2} \tag{7}$$

$$\frac{d}{dt}B_a = \tilde{\delta}_B B + \tilde{\eta}_e(t)K_2(E,R)B + \tilde{W}(B,t) - dB_a - f_M M B_a - f_{M_a} M_a B_a - f_{tD}(D_{ss} - D)B_a - f_D D B_a \tag{8}$$

$$\frac{d}{dt}B_n = dB_a - f_M M B_n - f_{M_a} M_a B_n - f_{tD}(D_{ss} - D)B_n - f_D D B_n. \tag{9}$$

$$\frac{d}{dt}G = R_0 - (G_0 + S_I I)G, \tag{10}$$

$$\frac{d}{dt}I = \sigma_I K_1(G, G_I)B - \delta_I I. \tag{11}$$

$$\frac{d}{dt}D = f_{tD}B_n(D_{ss} - D - tD) + f_{tD}B_n tD - b_{DE}ED - \mu_D D \tag{12}$$

$$\frac{d}{dt}tD = f_{tD}B_a(D_{ss} - D - tD) - f_{tD}B_n tD - b_{IR}RtD - \mu_D tD. \tag{13}$$

$$\frac{dE}{dt} = a_E\left(T_{naive} - E\right) + b_P\frac{DE}{\theta_D + D} - r_{am}E + b_E DEm - \mu_E ER, \tag{14}$$

$$\frac{dR}{dt} = a_R\left(T_{naive} - R\right) + b_P\frac{tDR}{\theta_D + tD} - r_{am}R + b_R tDEm - \mu_R ER, \tag{15}$$

$$\frac{dEm}{dt} = r_{am}(E + R) - (a_{Em} + b_E D + b_R tD)Em. \tag{16}$$

Model Parameters

Parameter name	Balb/c Mice	NOD Mice	Units	Description;Eq. number	Citation
J	5×10^4	5×10^4	cells ml^{-1} day^{-1}	Resting macrophage influx; Eq. (1)	[1]
k	0.4	0.4	day^{-1}	Macrophage deactivation rate; Eq. (1)-(2)	[1]
b	0.09	0.09	day^{-1}	Recruitment rate of macrophages by activated macrophages; Eq. (1)	[1]
c	0.1	0.1	day^{-1}	Macrophage egress rate; Eq. (1)	[1]
e_1	1×10^{-8}	1×10^{-8}	cells^{-1} day^{-1}	Effect of crowding on macrophages; Eq. (1)	[1]
e_2	1×10^{-8}	1×10^{-8}	cells^{-1} day^{-1}	Effect of crowding on active macrophages; Eq. (2)	[1]
f_M	$0.0623 \times 2 \times 10^{-5}$	$0.0623 \times 1 \times 10^{-5}$	ml cells^{-1} day^{-1}	Rate macrophages engulf necrotic and apoptotic β-cells; Eq. (1)-(2) and Eq. (8)-(9)	Modified from [1]
f_{M_a}	$0.0623 \times 5 \times 10^{-5}$	$0.0623 \times 1 \times 10^{-5}$	ml cells^{-1} day^{-1}	Rate activated macrophages engulf necrotic and apoptotic β-cells; Eq. (8)-(9)	Modified from [1]
α_B	0.0334	0.0334	day^{-1}	Rate β-cells are produced from glucose; Eq. (3)	[2]
G_{hb}	90	90	mg dl^{-1}	Glucose level of half-max β-cell production; Eq. (3)-(4)	[2]
δ_B	0.0167	0.0167	day^{-1}	β-cell death rate; Eq. (3) and Eq. (8)	[2]
Q_{panc}	0.194	0.194	ml	Volume of mouse pancreas	[3]
B_{conv}	2.59×10^5	2.59×10^5	cell mg^{-1}	β-cells per milligram	Uses β-cell count from [4]
η	0.02	0.02	day^{-1}	Rate at which T cells eliminate β-cells; Eq. (3) and Eq. (8)	Estimated here
s_E	1	1	ml cells^{-1}	Relative impact of effector T cells on β-cell death; Eq. (5)	Estimated here
s_R	36	36	ml cells^{-1}	Relative impact of regulatory T cells on β-cell death; Eq. (5)	Estimated here
α_e	.11	.11	day^{-1}	Rate of effector T cell avidity for β-cells; Eq. (6)	Estimated from [5]
β_e	21	21	day	Half-max value for T cell killing of β-cells; Eq. (6)	Estimated from [5]
d	0.50	0.50	day^{-1}	β-cell rate of necrosis; Eq. (8)-(9)	[1]
f_D	1.71×10^{-7}	1.71×10^{-7}	ml cells^{-1} day^{-1}	Rate DCs engulf β-cells; Eq. (8)-(9)	Computed, see Sect. 2.5
f_{tD}	1.19×10^{-6}	1.19×10^{-6}	ml cells^{-1} day^{-1}	Rate naive or tolerogenic DCs engulf β-cells; Eq. (8)-(9); Eq. (12-13)	Computed, see Sect. 2.5
D_{ss}	1×10^5	1×10^5	cells ml^{-1}	Steady-state DC population; Eq. (8)-(9), Eq. (12)-(13)	Estimated here
R_0	864	864	mg dl^{-1}	Basal rate of glucose production; Eq. (10)	[6]
G_0	1.44	1.44	day^{-1}	Rate of glucose decay; Eq. (10)	[6]
S_I	0.72	0.72	ml μU^{-1} day^{-1}	Rate of glucose elimination via insulin; Eq. (10)	[6]
G_I	$\sqrt{20000}$	$\sqrt{20000}$	mg dl^{-1}	Glucose level of half-max insulin production; Eq. (11)	[6]
σ_I	43.2	43.2	μU ml^{-1} day^{-1} mg^{-1}	Maximum rate of insulin production by β-cells; Eq. (11)	[6]
δ_I	432	432	day^{-1}	Rate of insulin decay; Eq. (11)	[6]
b_{DE}	0.487×10^{-5}	0.487×10^{-5}	ml cells^{-1} day^{-1}	Rate of elimination of DCs by effector T cells; Eq. (12)	[7]
b_{IR}	0.487×10^{-5}	0.487×10^{-5}	ml cells^{-1} day^{-1}	Rate of elimination of tDCs by regulatory T cells; Eq. (13)	Estimated same as b_{DE}
μ_D	0.51	0.51	day^{-1}	Rate of removal from pancreas for DC and tDC; Eq. (12)-(13)	Estimated from [7]

a_E	.1199	.1199	day^{-1}	Rate of initial expansion of naive T cells into effector T cells; Eq. (14)	[7]
a_R	.1199	.1199	day^{-1}	Rate of initial expression of naive T cells into regulatory T cells; Eq. (15)	Estimated same as a_E
T_{naive}	370	370	cells ml^{-1}	Density of naive T cells contributing to initial production of effector and regulatory T cells in the spleen; Eq. (14)-(15)	[7]
b_P	12	12	day^{-1}	Maximal expansion rate of effector and regulatory T cells due to DCs; Eq. (14)-(15)	[7]
r_{am}	0.01	0.01	day^{-1}	Reversion rate of effector and regulatory T cells to memory T cells; Eq. (14)-(16)	[7]
b_E	1×10^{-3}	1×10^{-3}	ml day cells^{-1}	Activation rate for effector T cells from memory T cells; Eq. (16)	Estimated from [7]
μ_E	2×10^{-6}	2×10^{-6}	day^{-1}	Rate of effector T cell removal due to regulatory T; Eq. (14)	Estimated here
θ_D	2.12×10^5	2.12×10^5	day^{-1}	DC value for half-maximal effector T cell expansion; Eq. (14)-(15)	Estimated from [7]
b_R	1×10^{-3}	1×10^{-3}	ml day cells^{-1}	Activation rate for regulatory T cells from memory T cells; Eq. (15)-(16)	Estimated same as b_E
μ_R	2×10^{-6}	2×10^{-6}	day^{-1}	Rate of regulatory T cell removal due to effector T; Eq. (15)	Estimated same as μ_E
a_{Em}	0.01	0.01	day^{-1}	Death rate of memory T cells; Eq. (16)	[7]

Table 1: Model parameters.

References

[1] Marée AF, Kublik R, Finegood DT, Edelstein-Keshet L. Modelling the onset of Type 1 diabetes: can impaired macrophage phagocytosis make the difference between health and disease? Philosophical Transactions of the Royal Society of London A: Mathematical, Physical and Engineering Sciences. 2006;364(1842):1267–1282.

[2] Graham E. Mathematical Models of Mechanisms Underlying Long-term Type 2 Diabetes Progression [dissertation]. University of Utah. Salt Lake City, UT; 2012.

[3] Paredes JL, Orabi AI, Ahmad T, Benbourenane I, Tobita K, Tadros S, et al. A non-invasive method of quantifying pancreatic volume in mice using micro-MRI. PLoS ONE. 2014;9.

[4] Chintinne M, Stangé G, Denys B, Ling Z, In 't Veld P, Pipeleers D. Beta cell count instead of beta cell mass to assess and localize growth in beta cell population following pancreatic duct ligation in mice. PLoS One. 2012;7.

[5] Li A, Chen J, Hattori M, Franco E, Zuppan C, Ojogho O, et al. A therapeutic DNA vaccination strategy for autoimmunity and transplantation. Vaccine. 2010;28(8):1897 – 1904. doi:https://doi.org/10.1016/j.vaccine.2009.10.090.

[6] Topp B, Promislow K, Devries G, Miura RM, T Finegood D. A model of β-cell mass, insulin, and glucose kinetics: pathways to diabetes. Journal of theoretical biology. 2000;206(4):605–619.

[7] Ludewig B, Krebs P, Junt T, Metters H, Ford NJ, Anderson RM, et al. Determining control parameters for dendritic cell-cytotoxic T lymphocyte interaction. European Journal of Immunology. 2004;34(9):2407–2418.
